# Non-targeted transcriptomic effects upon thyroid irradiation: similarity between in-field and out-of-field responses varies with tissue type

**DOI:** 10.1038/srep30738

**Published:** 2016-10-25

**Authors:** Britta Langen, Nils Rudqvist, Johan Spetz, John Swanpalmer, Khalil Helou, Eva Forssell-Aronsson

**Affiliations:** 1 Department of Radiation Physics, Institute of Clinical Sciences, Sahlgrenska Cancer Center, Sahlgrenska Academy, University of Gothenburg, Gothenburg, Sweden; 2 Department of Applied Physics, Chalmers University of Technology, Gothenburg, Sweden; 3 Department of Medical Physics and Biomedical Engineering, Sahlgrenska University Hospital, Gothenburg, Sweden; 4 Department of Oncology, Institute of Clinical Sciences, Sahlgrenska Cancer Center, Sahlgrenska Academy, University of Gothenburg, Gothenburg, Sweden

## Abstract

Non-targeted effects can induce responses in tissues that have not been exposed to ionizing radiation. Despite their relevance for risk assessment, few studies have investigated these effects *in vivo*. In particular, these effects have not been studied in context with thyroid exposure, which can occur e.g. during irradiation of head and neck tumors. To determine the similarity between in-field and out-of-field responses in normal tissue, we used a partial body irradiation setup with female mice where the thyroid region, the thorax and abdomen, or all three regions were irradiated. After 24 h, transcriptional regulation in the kidney cortex, kidney medulla, liver, lungs, spleen, and thyroid was analyzed using microarray technology. Thyroid irradiation resulted in transcriptional regulation in the kidney medulla and liver that resembled regulation upon direct exposure of these tissues regarding both strength of response and associated biological function. The kidney cortex showed fewer similarities between the setups, while the lungs and spleen showed little similarity between in-field and out-of-field responses. Interestingly, effects were generally not found to be additive. Future studies are needed to identify the molecular mechanisms that mediate these systemic effects, so that they may be used as targets to minimize detrimental side effects in radiotherapy.

Radiation biology is based on the paradigm that DNA double-strand break induction is the hallmark event upon ionizing radiation (IR) exposure that determines cellular outcome^1^. Observations of non-targeted effects in experimental and clinical settings have challenged this paradigm, which has been frequently reviewed (and discussed) in recent years^2^,^3^,^4^,^5^,^6^,^7^,^8^. Based on the context, non-targeted effects are also termed ‘bystander effect’ for unirradiated cells in the vicinity of irradiated cells, ‘abscopal’ or ‘out-of-field’ effects in external irradiation, or ‘long-range bystander effects’ or ‘systemic effects’ when regarding a complex physiological setting^2^,^3^,^4^,^5^,^6^,^7^,^8^. The question has been raised whether non-targeted effects represent a new paradigm in radiation research or simply the fact of biological complexity^8^. It has also been proposed that the frequency of reported clinical abscopal effects may increase due to the increasing use of stereotactic ablative body radiation therapy and hypofractionation techniques^9^. While the underlying mechanisms of *in vivo* abscopal bystander effects remain elusive^3^, it has been reasoned that normal tissue responses in radiotherapy are highly complex and that the degree of non-targeted effects are not only related to the irradiated volume or physical irradiation parameters^8^,^10^. Non-targeted effects are considered relevant for radiation therapy, both for use as a novel therapeutic target and for improvement of risk assessment^4^,^8^,^11^,^12^,^13^,^14^,^15^,^16^.

Recent work demonstrated non-targeted responses in lung and liver tissues upon partial body irradiation (5 Gy) of the lower abdomen using 300 keV X-rays^17^,^18^. While IR exposure from external beam radiation therapy or X-rays may be localized, exposure from i.v. administered radiopharmaceuticals in radionuclide therapy is systemic. In clinical practice, free radionuclides and radiolabeled carrier molecules are used for diagnostics and therapy. During metabolism or degradation of radiolabeled carrier molecules, radionuclides can be liberated from the carrier molecules and accumulate in normal tissue. In the case of ^131^I and ^211^At, the thyroid gland is a risk organ due to high uptake similar to that of stable iodide^19^,^20^,^21^,^22^,^23^,^24^. However, basically all tissues show uptake of these radionuclides, although to a much lesser degree that generally varies between tissues^25^.

Knowledge of normal tissue response to low-dose exposure from radionuclides *in vivo*, however, is still scarce. Another issue that has not been sufficiently addressed in both the experimental and clinical setting are systemic effects between tissues. Specifically, differential uptake of ^131^I or ^211^At creates a concomitant low-dose and high-dose exposure setting within the body. In this case, responses in the kidneys, liver, lungs, and spleen would not only be subject to (very) low absorbed dose from radionuclide decay in the tissue, but may also respond to IR-induced effects in the thyroid, since the thyroid gland regulates metabolic function throughout the body. The nature of potential systemic effects could be ‘physiological’ due to disrupted hormone regulation in the thyroid, or radiation-associated in the sense of ‘long-range non-targeted effects’.

In previous studies using BALB/c nude mice as a model system, we observed distinct similarities in genome-wide transcriptional regulation between the thyroid and various non-thyroid tissues despite large differences in absorbed dose levels from ^211^At^26^,^27^. We hypothesized that observed responses in non-thyroid tissues resulted, in part, from systemic factors originating from the dominantly irradiated thyroid gland^27^. Differential regulation of thyroid hormone (TH)-responding genes even at very low absorbed doses in non-thyroid tissues supported this hypothesis^28^,^29^.

The aim of this study was to confirm systemically induced transcriptional regulation in non-thyroid tissues after IR exposure of the thyroid. An external partial body irradiation setup was used to preclude differential exposure throughout the body as was the case in previous studies using i.v. administered radionuclides, i.e. to exclude concomitant IR-induced regulation in each tissue from regulation induced by thyroid-dependent systemic effects.

## Results

### Total transcriptional regulation in response to partial body irradiation

The total number of transcripts and corresponding genes that were significantly regulated 24 h after exposure are shown for each tissue and each irradiation setup in Table 1. Microarray data was validated using the quantitative real-time polymerase chain reaction (QPCR) assay. (For QPCR results, please refer to Supplementary Table S1.) Regarding the number of regulated transcripts, an additive effect between the partial body irradiation setups was not observed in the kidney cortex, kidney medulla, lungs, or thyroid, i.e. the result for group C was not the “sum” of the results for group A and B. The spleen might suggest an additive effect in this context, but the effect on upregulation was not consistent, since the number of upregulated transcripts decreased distinctly upon combined irradiation (group C) compared with non-thyroid tissue irradiation (B). In contrast, a potential trend towards an additive effect was seen in the liver, but it should be noted that the total number of regulated transcripts upon combined irradiation (group C) was somewhat higher than a strict addition would suggest.

In the kidney cortex, kidney medulla, and liver, irradiation of the thyroid (group A) resulted in an overall transcriptional regulation that lay on a similar–if not higher–level as when only non-thyroid tissues (group B) were irradiated. In contrast, few transcripts were regulated in the lungs and spleen when only the thyroid was irradiated (group A). The kidney medulla showed the highest number of regulated transcripts (252–274 transcripts) at all irradiation setups, which was at least twice as many as observed for the other tissues. Interestingly, the kidney medulla also showed a decidedly higher number of regulated transcripts than the thyroid when only the thyroid was irradiated (group A). Comparing exclusive irradiation of non-thyroid tissues (group B) with combined irradiation of all tissues (group C), the total number of regulated transcripts was similar in the kidney cortex (89 vs. 84 transcripts), kidney medulla (274 vs. 259 transcripts), and spleen (133 vs. 135 transcripts), but the ratio between up- and downregulation differed distinctly between both irradiation setups. In the liver and lungs, irradiation of non-thyroid tissues (group B) or all tissues (group C) showed larger differences, however. The highest number of regulated transcripts in the liver occurred in group C (132 transcripts), whereas the lungs showed a distinct peak in group B (70 transcripts); up- and downregulation also differed in these tissues. In the thyroid, exclusive irradiation (group A) resulted in a distinctly higher response (115 transcripts) than irradiation in combination with the other tissues (25 transcripts, group C); however, the latter was on a similar level as transcript regulation in the absence of thyroid irradiation (17 transcripts, group B).

In general, the extent of up- and downregulation in each tissue differed between irradiation setups. The only notable exception was observed in the kidney medulla where irradiation of thyroid (group A) and irradiation of non-thyroid tissues (group B) resulted not only in a similar number of regulated transcripts, but also in a similar ratio (ratio: 4.3–5.0) of up- vs. downregulation. Interestingly, this ratio decreased distinctly (ratio: 1.2) when all tissues were irradiated (group C), while the total number of regulated transcripts remained similar.

### Gene regulation in a tissue shared across irradiation setups

**Figure 1:**
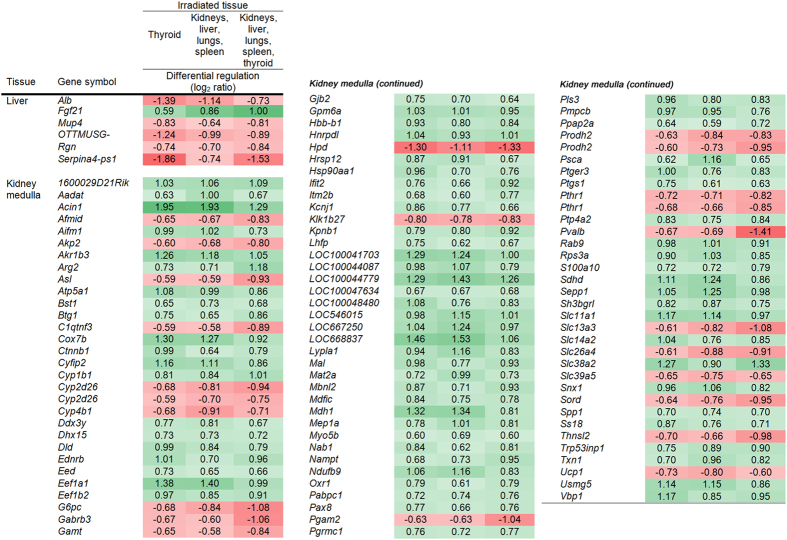
Transcripts regulated at all irradiation setups. The figure shows the transcripts that were regulated after 24 h across all irradiation setups in the liver and kidney medulla with respective log_2_ ratios for each irradiation setup (irradiated tissue(s), i.e. groups (A–C). In the kidney cortex, lungs, spleen, and thyroid, no transcripts were continuously regulated across all irradiation setups. Positive numbers indicate upregulation (green), negative numbers indicate downregulation (red).

Comparison analysis identified genes in the liver and kidney medulla that were regulated across all irradiation setups (Fig. 1). For further information regarding probe ID and transcript ID of respective transcripts, please refer to Supplementary Fig. S1. Shared transcript regulation across all irradiation setups was not observed in the kidney cortex, lungs, spleen, and thyroid. In the liver, 6 genes (6 transcripts) were identified, with 5 thereof being continuously downregulated. In the kidney medulla, 98 genes (101 transcripts) were identified which made up around 40% of the total number of regulated transcripts (*cf.* Table 1). In contrast to the liver, the majority of genes (77 of 98) were continuously upregulated. In both tissues, all genes maintained the same direction of regulation irrespective of irradiation setup. Differential regulation was on a comparatively low level in both tissues with maximum log_2_ ratios of 1.95 and −1.86 up- and downregulation, respectively. In total, these genes were regulated in a similar fashion irrespective of the irradiated region, i.e. whether or not the tissue was exposed to ionizing radiation.

**Figure 2:**
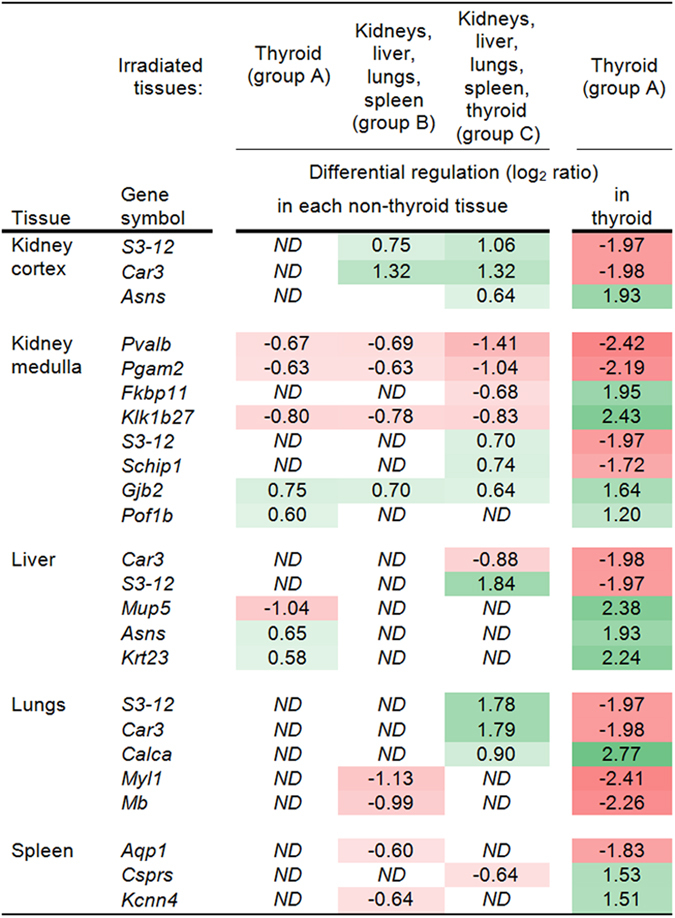
Shared transcript regulation among thyroid and other tissues at differential exposures. The figure shows the log_2_ ratios of transcripts that were regulated after 24 h in a given non-thyroid tissue for at least one irradiation setup (groups (A–C)) and that were also regulated in the thyroid upon thyroid irradiation (group A). Positive numbers indicate upregulation (green), negative numbers indicate downregulation (red); ND, none detected.

The similarity between thyroid-specific gene regulation and regulation in the investigated non-thyroid tissues was analyzed between different exposure setups (Fig. 2). For further information regarding probe ID and transcript ID of respective transcripts, please refer to Supplementary Fig. S2. For each non-thyroid tissue, data from each irradiation setup (groups A–C) were compared individually with thyroid data from group A. As such, the analysis gave information on the shared gene regulation between thyroid tissue upon thyroid irradiation and regulation in non-thyroid tissues for each irradiation setup. Few similarities in specific gene regulation were observed in this comparison analysis: in the kidney cortex, lungs, and spleen, no genes were shared with the thyroid when only thyroid was irradiated (group A); in the kidney medulla and liver, 5 and 3 genes were shared with thyroid in this setup (group A), respectively. Regarding irradiation of non-thyroid tissues (group B), all non-thyroid tissues except for the liver shared at least 2 regulated genes with the thyroid (group A). When all tissues were irradiated (group C), the number of transcripts shared with the thyroid increased somewhat in all tissues except for the spleen. Log_2_ ratios of shared transcripts were generally low in the non-thyroid tissues but somewhat higher in the thyroid. The only genes that were regulated in a non-thyroid tissue across all irradiation setups (groups A–C) and were also regulated in the thyroid (groups A) were identified in the kidney medulla: *Pvalb* and *Pgam2* (continuously downregulated), *Gjb2* (continuously upregulated), and *Klk1b27* (continuously downregulated in the kidney medulla, but upregulated in the thyroid).

### Regulation of IR- and TH-associated signature genes

**Figure 3:**
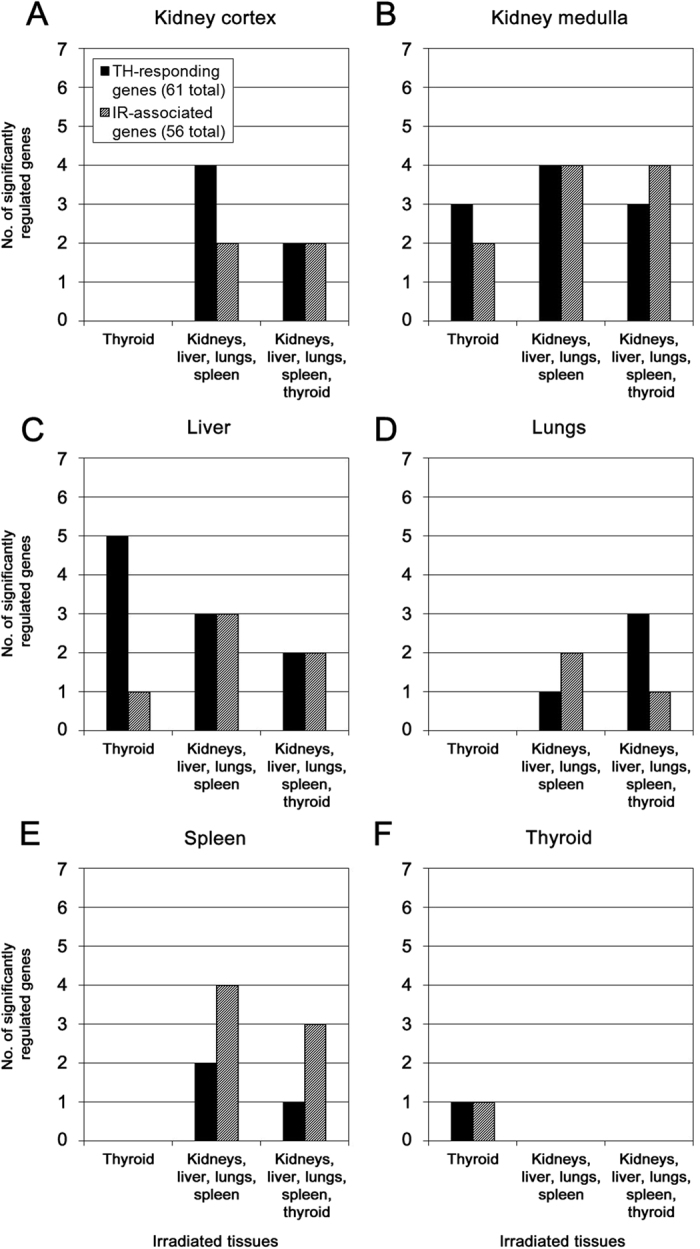
Transcriptional regulation in IR-associated and TH-responding gene signatures. The number (no.) of significantly regulated genes in either signature (thyroid hormone (TH)-responding genes, black; ionizing radiation (IR)-associated genes, gray) is shown for the kidney cortex (**A**), kidney medulla (**B**), liver (**C**), lungs (**D**), spleen (**E**), and thyroid (**F**). Regulation was measured at 24 h following irradiation of the collum (thyroid irradiated), abdomen & thorax (kidneys, liver, lungs, and spleen irradiated), or collum & abdomen & thorax (kidneys, liver, lungs, spleen, and thyroid irradiated). Please refer to Supplementary Tables S2 and S3 for further information on gene symbol, transcript ID, and log_2_ ratio of regulated genes.

The number of regulated genes in the IR-associated and TH-responding gene signature generally differed between the tissues at the same irradiation setup (Fig. 3). For information on gene name, probe and transcript ID, and log_2_ ratio of regulated IR-associated and TH-responding genes, please refer to Supplementary Tables S2 and S3, respectively. Interestingly, regulation of both gene signatures was observed in the kidney medulla (Fig. 3B) and in the liver (Fig. 3C) in the absence of direct ionizing radiation exposure, i.e. when only the thyroid was irradiated (group A). In contrast, signature gene regulation was not detected in the kidney cortex (Fig. 3A), lungs (Fig. 3D), and spleen (Fig. 3E) for that condition (group A). The largest overall response was observed in the kidney medulla followed by the liver and kidney cortex.

IR-associated signature genes were detected in all investigated non-thyroid tissues upon irradiation (groups B–C), as were TH-responding signature genes. The average number of regulated genes for either signature was higher in the kidney cortex, kidney medulla, and liver than in the lungs and spleen. A clear dominance of a signature was only observed in the liver for TH-responding genes (specifically group A) and in the spleen for IR-associated genes (groups B–C), while the kidney cortex indicated a trend towards TH-responding genes upon irradiation of non-thyroid tissues (group B). In the kidney medulla, the number of regulated genes was comparatively high but on the same level for each signature irrespective of irradiation setup. The lungs exhibited the lowest overall number of regulated signature genes among non-thyroid tissues and showed the lowest response in IR-associated genes (groups B–C). Interestingly, regulation of TH-responding genes in the lungs outweighed IR-associated genes when all tissues were irradiated, which was not observed among the other tissues for that condition (group C). In the thyroid (Fig. 3F), signature gene regulation was only observed when the tissue was irradiated (group A) but not upon irradiation of non-thyroid tissues (group B) or when the thyroid was irradiated in combination with non-thyroid tissues (group C). Compared with the non-thyroid tissues, the response was low with only one regulated gene for each signature.

Often, the same genes were regulated when several signature genes were detected for two or more irradiation setups (*cf*. Supplementary Tables S2 and S3). In general, average log_2_ ratios (i.e. absolute values of up- and down regulation) lay on a similar level for IR-associated and TH-responding signature genes. The kidney cortex, kidney medulla, and liver showed the lowest average log_2_ ratios for either signature, i.e. 1.00, 0.86, and 1.05, respectively, for IR-associated genes and 0.85, 0.94, and 0.93, respectively, for TH-responding genes. Although the lungs and spleen had a lower response in the number of regulated signature genes, both tissues showed slightly higher average log_2_ ratios for both signatures (IR-associated genes, average log_2_ ratio: 1.20 (lungs) and 1.34 (spleen); TH-responding genes, average log_2_ ratio: 1.09 (lungs) and 1.75 (spleen)). Thyroid displayed the lowest number of regulated signature genes but also the highest log_2_ ratios for differential regulation: the IR-associated gene *Gjb2* was (up-)regulated with a log_2_ ratio of 2.19 (average of two probes) and the TH-responding gene *Atp2a1* was (down-)regulated with a log_2_ ratio of (−2.41).

### Regulation profiles of associated cellular function

**Figure 4:**
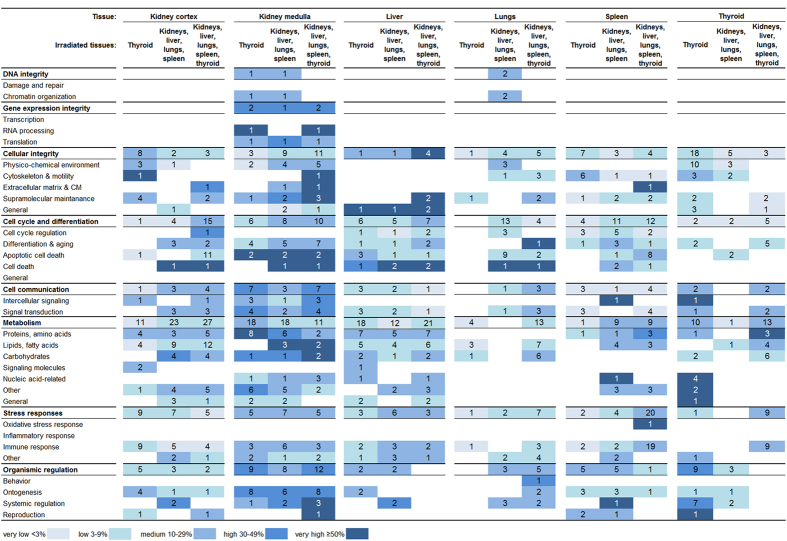
Response profiles of enriched biological processes categorized after cellular function. Significantly regulated transcripts were enriched for biological processes which were grouped in respective categories and subcategories of higher level cellular function. Regulation was measured 24 h following irradiation of collum (thyroid irradiated), abdomen & thorax (kidneys, liver, lungs, and spleen irradiated), or collum & abdomen & thorax (kidneys, liver, lungs, spleen and thyroid irradiated). The percentage of scored vs. filtered transcripts is illustrated as very low < 3% (very light blue), low 3–9% (light blue), medium 10–29% (blue), high 30–49% (dark blue), and very high ≥ 50% (very dark blue). Numbers indicate the sum of regulated biological processes within a category or subcategory.

Categorization of enriched biological processes revealed similarities and differences between the irradiation setups with regard to cellular function (Fig. 4). In most tissues, no effect on *DNA integrity* and *gene expression integrity* was observed.

The highest level of regulation and the largest diversity across categories were observed in the kidney medulla. This tissue also showed the highest degree of similarity between the irradiation setups, meaning with regard to both regulated subcategories and intensity of regulation. In the kidney medulla, all main categories of cellular function were regulated at basically every irradiation setup, i.e. the only exception was found for *DNA integrity* when all tissues were irradiated (group C). It should be noted, however, that respective regulations of *DNA integrity* in groups A–B consisted of only one biological process. Between irradiation of thyroid (group A) and irradiation of non-thyroid tissues (group B), the highest similarity was observed for *metabolism, stress responses*, and *organismic regulation*. The other categories also exhibited similar trends for several subcategories. Distinct dissimilarity was only observed for *cellular integrity,* where irradiation of all tissues (group C) resulted in a distinctly stronger regulation (regarding number of subcategories, biological processes, and intensity of regulation) than irradiation of thyroid alone (group A). Despite the diverse regulation across categories, several cellular functions were not regulated at any irradiation setup on the subcategorical level in the kidney medulla, i.e. *(DNA) damage and repair*, *transcription*, *cell cycle regulation*, *general cell cycle and regulation*, *signaling molecules*, *oxidative stress response*, *inflammatory responses*, and *behavior*.

In the liver, cellular functions were regulated with less diverse patterns compared with the kidney medulla, i.e. fewer subcategories were regulated, yet the patterns also showed distinct similarities between all irradiation setups. Similar regulation patterns were seen in the categories *cellular integrity*, *cell cycle and differentiation*, *cell communication*, and *stress responses*, and to a somewhat lesser degree in *metabolism*.

In the kidney cortex, the diversity and intensity of responses resembled the liver. However, the regulation patterns upon thyroid irradiation (group A) were less similar to non-thyroid tissue irradiation (group B) or to combined irradiation of all tissues (group C). Between irradiation of non-thyroid tissues (groups B) and all tissues (group C), distinctly similar regulation patterns were observed in *metabolism* and *stress responses,* and to some extent in *cell communication*.

In the lungs and spleen, regulation patterns were distinctly dissimilar between all irradiation setups. Similar responses were only observed in the spleen between groups B and C in *cell cycle and differentiation* and *metabolism*. Also, regulation patterns were considerably less diverse compared with the kidney medulla and spleen, i.e. fewer subcategories were regulated.

The thyroid showed the highest level of regulation when thyroid was irradiated (group A), specifically in *cellular integrity* and *organismic regulation*, compared with the other exposure setups. Regarding *metabolism*, the total number of regulated processes was similarly high upon thyroid irradiation (group A, 10 processes) and upon irradiation of all tissues (group C, 13 processes), but the distribution on the subcategorical level differed distinctly. The lowest response in the thyroid was seen when only non-thyroid tissues were irradiated (group B). Interestingly, regulation patterns after irradiation of thyroid alone (group A) showed few similarities with thyroid irradiation in combination with non-thyroid tissues (group C).

### Predicted pathway regulation of diseases and biological functions

**Figure 5:**
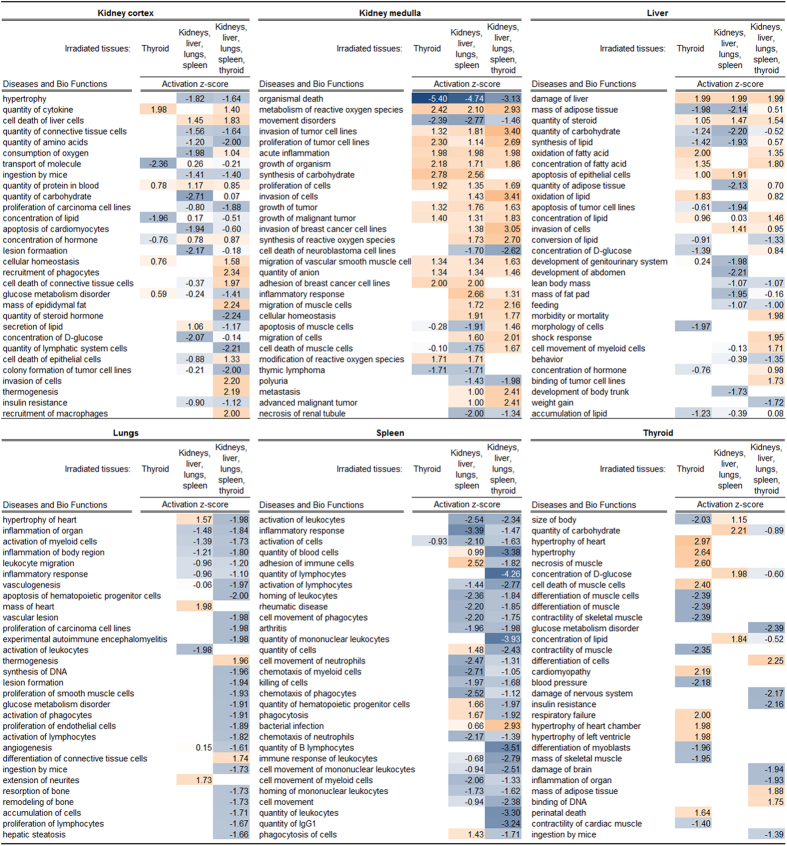
Pathway analysis of diseases and functions shared between irradiation conditions. IPA (Ingenuity^®^ Systems) was used to analyze regulation of pathways associated with diseases or biological functions (termed *Diseases and Bio Functions*) after 24 h following irradiation. Results for each tissue were obtained by comparison analysis across all irradiation setups. Calculated z-scores for activation (orange) or inhibition (blue) are shown for the 30 highest-ranked diseases and functions. For complete lists of regulated *Diseases and Bio Functions* in the kidney cortex, kidney medulla, liver, lungs, spleen, and thyroid, please refer to Supplementary Tables S4–S9, respectively.

Pathway analysis predicted the activation (or inhibition) state of associated diseases and biological functions. The thirty highest-ranked diseases and biological functions according to z-score are shown (Fig. 5). Although significance of activation state (|z-score| > 2) was not achieved for a large proportion of these pathways in the kidney cortex, liver, and lungs, the obtained data did indicate trends towards similarity or difference of responses between the irradiation setups. Please refer to Supplementary Tables S4–S9 for complete lists of generated diseases and functions for the kidney cortex, kidney medulla, liver, lungs, spleen, and thyroid, respectively.

The highest degree of similarity–here meaning both activation state (activation or inhibition) and strength of prediction (activation z-score) between all irradiation setups was seen in the kidney medulla. Eighteen of these pathways identified upon thyroid irradiation (group A) were regulated with the same predicted activation state and, in most cases, similar z-score as when only non-thyroid tissues were irradiated (group B). Among these pathways, activation was more frequent than inhibition. Irradiation of all tissues (group C) showed a higher degree of similarity with irradiation of non-thyroid tissues (group B), although around one-third of the pathways were also predicted to be regulated in a similar manner upon thyroid irradiation (group A).

The liver showed less similarity in pathway regulation between the irradiation setups than the kidney medulla. Around one-third of the pathways were shared between thyroid irradiation (group A) and non-thyroid tissue irradiation (group B), which was somewhat lower than the number of pathways shared between non-thyroid tissue irradiation (group B) and combined irradiation of all tissues (group C). Only a minority of these pathways were shared between all irradiation setups. The activation state did not show a distinct tendency towards activation or inhibition as was observed in the kidney medulla.

In the kidney cortex, only five pathways were shared between all irradiation setups and most instances thereof did not show a significant activation (or inhibition) state. Notably, only few regulation instances with weak prediction strength were observed upon thyroid irradiation (group A). In contrast, around two-thirds of these thirty pathways were shared between irradiation of non-thyroid tissues (group B) and in combination with thyroid (group C). Although a tendency towards pathway inhibition was somewhat more frequent than activation, few of these instances had significant prediction strength.

In the lungs, pathway regulation was not predicted upon thyroid irradiation (group A; *cf.* Supplementary Table S7). Shared regulation between irradiation of non-thyroid tissues (group B) and irradiation of all tissues (group C) was distinctly less frequent than observed in the kidney cortex, kidney medulla, and liver. Although the vast majority of instances did not achieve significant prediction strength, a distinct trend towards inhibition was observed upon irradiation of all tissues (group C).

The regulation pattern in the spleen showed similarity to the lungs: pathway regulation upon thyroid irradiation was basically not detected (barring two exceptions, *cf.* Supplementary Table S8) and most regulation instances showed inhibition or a trend towards inhibition. In contrast to the lungs, however, the vast majority of pathways were shared between irradiation of non-thyroid tissues (group B) and irradiation of all tissues (group C). Moreover, the spleen generally showed stronger prediction strength and a higher number of shared pathways compared with lungs (*cf.* Supplementary Tables S7 and S8).

In the thyroid, the highest frequency of pathway regulation among the thirty highest ranked pathways was seen upon irradiation of the tissue (group A). In contrast, the frequency of pathway regulation upon exclusive irradiation of non-thyroid tissues was very low (group B). Only few identified pathways were shared between irradiation of thyroid (group A) and thyroid irradiation in combination with the other tissues (group C), also showing large discrepancy in prediction strength. Considering all identified pathways in the thyroid, activation (or trend towards activation) was somewhat more frequent than inhibition (or trend towards inhibition) (*cf.* Supplementary Table S9).

## Discussion

Partial body irradiation of the thyroid gland resulted in transcriptional regulation in the kidney medulla and liver that resembled responses to irradiation of the thorax and abdomen. In the kidney cortex, the total number of regulated transcripts was rather similar between the irradiation setups, but the transcript-associated cellular functions displayed a higher degree of heterogeneity. The lungs and spleen exhibited basically no transcriptional regulation in the absence of direct tissue irradiation. In summary, out-of-field responses on the transcriptional level resembled in-field responses to varying extent depending on the type of tissue. This tendency was also in agreement with the tissue-specific tendency of TH-responding signature gene regulation. The regulation of TH-responding signature genes in the absence of thyroid irradiation, which was seen in all non-thyroid tissues to varying extent, might be due to down-stream effects of IR-induced gene regulation in each tissue, or–in certain part–might result from thyroid-dependent stimuli induced by scattered photons, which might have occurred upon large-field irradiation of the thorax and abdomen. It should be pointed out that the overall transcriptional response, as well as individual gene regulation and functional association, were studied in female mice and not in a cohort including both sexes. Female BALB/c nude mice were chosen to match the respective tumor xenograft model that is routinely used in therapeutic pre-clinical studies. As such, before validation for both sexes, these results should not be generalized as sex-independent.

A pronounced effect on transcriptional regulation in thyroid tissue was expected upon exclusive irradiation of the thyroid with 2 Gy, which was in agreement with the 115 significantly detected transcripts. The cause of transcriptional regulation in the thyroid in the absence of irradiation remained a point of speculation. A possible explanation may be that the thyroid gland was subjected to absorbed dose in the mGy range at the border of the irradiated volume. In this scenario, low-level exposure may well have resulted in regulation of the 17 detected transcripts. Surprisingly, the total number of regulated transcripts in the thyroid lay on a similar level upon irradiation of non-thyroid tissues and upon combined irradiation of all tissues. On a similar note, the overall transcriptional regulation was decidedly lower in the thyroid upon combined irradiation with non-thyroid tissues than upon exclusive thyroid irradiation. In other words, absorbed dose to thyroid was found to not be the single factor influencing the strength of total transcriptional regulation in thyroid tissue. It is possible that transcriptional regulation in the thyroid underlay different mechanisms when a large volume of the body was irradiated. Whether this response was related specifically to one (or more) of the investigated non-thyroid tissues remained a point of speculation. It may be possible to further investigate this point with exclusive irradiation of each non-thyroid tissue and subsequent analysis of induced transcriptional effects in thyroid tissue.

The systemic context between tissues *in vivo* has consequences for analyzing normal tissue responses to ionizing radiation when the tissue of interest underlies stimuli from another tissue that was (also) exposed to ionizing radiation. Barring variation between different biological endpoints, a quantified response would thus not be a monocausal effect resulting from ionizing radiation exposure, but may represent–to a certain extent–an effect induced by another tissue. The extent of similarity was analyzed by means of shared gene regulation across all irradiation setups, as well as data enrichment for functional pathway analysis. The kidney medulla showed a large number of genes that were regulated in a highly similar fashion at all irradiation setups; such a finding was also demonstrated in the liver, although the number of genes was comparatively low.

This finding raises the question how gene regulation in the kidney medulla should be interpreted upon in-field exposure when a large percentage of genes were also regulated when the tissue was out-of-field, i.e. when only the thyroid was irradiated. The analytical dilemma becomes even more apparent when considering that combined irradiation of the thyroid and non-thyroid tissues resulted in a distinctly similar regulation pattern in that gene set as was observed for the other two irradiation setups. The change extent of individual transcript regulation is a suitable measure to evaluate potential additive or even synergistic effects in the biological response. However, even log_2_ change values in this gene set for kidney medulla were basically on the same level and did not indicate additive effects between irradiations setups.

Furthermore, this finding has implications for biomarker discovery and risk assessment in radiotherapy when the thyroid is a risk organ, since down-stream responses in other organs, in particularly in the kidneys and liver, can also be expected. Understanding the complexity of *in vivo* responses, in this case regulatory dependency between tissues, offers a new venue for estimating and counteracting side effects that may otherwise have appeared intangible or unrelated in non-targeted tissues. As such, these genes constitute a promising panel of biomarker candidates for assessing side-effects in non-irradiated tissues. Individual genes may even serve as molecular targets to modulate pathways to decrease detrimental tissue responses. However, the dose-response of this gene set needs to be assayed in future studies and longitudinal studies are necessary to link early transcriptomic effects to long-term tissue health status.

In case of the thyroid gland, the physiological systemic context may appear obvious; however, to the best of our knowledge, the impact of the irradiated thyroid gland on non-thyroid tissue gene regulation has been suggested but not been demonstrated before^27^. Overall, considering all analytical endpoints and the extent of responses in the non-thyroid tissues upon thyroid irradiation (group A), the study demonstrated ‘relative thyroid dependence’ in transcriptional responses: the kidney medulla showed a high degree of ‘thyroid dependence’, the liver showed a high-to-medium degree of ‘thyroid dependence’, and the kidney cortex showed a medium degree of ‘thyroid dependence’. On the other hand, basically no ‘thyroid dependence’ was observed in the spleen. In the lungs, ‘thyroid dependence’ was not seen upon thyroid irradiation (group A), but an impact from thyroid irradiation was observed when non-thyroid tissues were irradiated in combination with the thyroid (*cf.* lungs group B and C, Figs 4 and 5).

Interestingly, the biological consequence of ‘thyroid dependence’ appeared to be rather different for kidney medulla and liver. In the kidney medulla, the biological functions with highest similarity and activation z-score were associated mainly with immune responses and tumor proliferation. In the liver, in contrast, the highest ranking functions were associated with liver-related metabolic processes. This finding may indicate that non-targeted responses can manifest in disease-related processes or tissue-specific metabolic processes depending on the type of tissue.

The demonstrated phenomenon may be denominated as a ‘physiological systemic effect’, or a ‘physiological long-range non-targeted effect’. Nevertheless, systemically acting factors may also result from mechanisms exclusively induced by ionizing radiation effects in the thyroid, i.e. additional factors that are to be distinguished from thyroid-specific signaling molecules such as thyroid hormones. Numerous types of non-targeted or bystander responses have been investigated and mechanisms for mediating these responses have been proposed^6^,^7^,^30^,^31^,^32^,^33^. The two main mechanisms of bystander responses are through gap junctions for cells in direct contact^8^,^34^,^35^ and through release of soluble factors^8^,^13^. The nature of long-range factors that mediated the observed out-of-field responses in this setting could not be deduced from microarray data. Likewise, potential ‘cross-talk’ between the kidneys, liver, lungs and spleen, which might have occurred in response to thyroid irradiation or upon IR exposure, could not be assessed.

In conclusion, this study demonstrated long-range non-targeted responses in the kidney cortex, kidney medulla, and liver upon irradiation of the thyroid region. The quantity and associated biological quality of transcriptional regulation in these tissues showed distinct similarities between in-field and out-of-field setups, and moreover, the extent of similarities varied with type of tissue. Further studies are needed to investigate the molecular mechanisms through which out-of-field effects occurred in the kidneys and liver–and why they did not occur in a similar fashion in the lungs and spleen. Biological variables such as sex, age, or species (strain) may influence these effects and need to be considered as well. Another aspect to be addressed is the temporal dependence of long-range non-targeted responses, meaning when a non-targeted effect occurs in a distant tissue, how long it persists, and if responses would e.g. depict a peak or saturation effect. The dependence on absorbed dose, dose rate, and radiation quality also needs to be analyzed in detail if systemic effects are to be used for risk assessment in radiotherapy.

## Methods

### Irradiation of animals

Fourteen female BALB/c nude mice (CAnN.Cg-Foxn1nu/Crl; Charles River, Salzfeld, Germany) were aged to four months and had access to water and (autoclaved) food *ad libitum*. The study protocol was approved by the Ethical Committee for Animal Research at University of Gothenburg, Gothenburg, Sweden (Permit Number: 362/12). All animal procedures were carried out in accordance with the ethical approval. Body measurements were performed on each animal using a caliper in order to assure similar geometry for irradiation planning. Distances were measured from nose to thyroid and from shoulder to base of the tail, as well as ventral-dorsal neck thickness, ventral-dorsal body thickness, and transversal body width.

**Figure 6:**
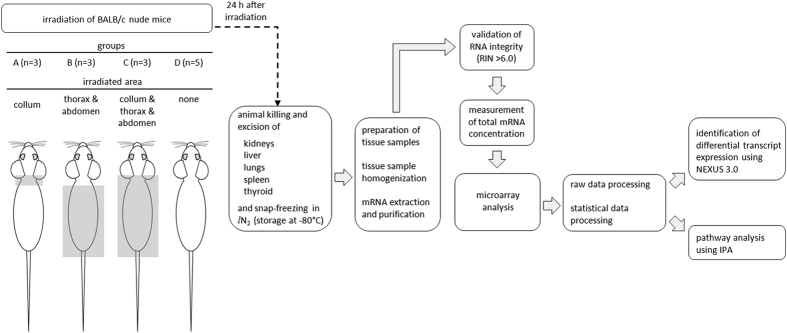
Illustration of workflow. The illustration shows the different irradiation setups and the main steps of subsequent sample preparation and data analysis.

For the irradiation, a linear accelerator (Varian Medical Systems; Palo Alto, CA, USA), with 4 MV nominal photon energy and a dose rate of 2.3 Gy/min was used. After anesthetization with an i.p. injection of Ketaminol^®^ vet. (Intervet AB; Sweden) and Domitor^®^ vet. (Orion Pharma Animal Health; Sweden), the mice were placed on their stomach in a prone position, head to gantry, on a polystyrene bed. The animals were subdivided into four groups (A–D) according to geometric similarity described above. The irradiation setup for each group is illustrated in Fig. 6. In group A (n = 3), only the thyroid region, i.e. collum, was irradiated and the radiation field at the isocenter (1,000 mm) was 10 × 10 mm. In group B (n = 3), the thorax and the abdomen were irradiated (field length and width: 60 × 50 mm). In group C (n = 3), the thyroid region (collum) as well as the thorax and abdomen were irradiated (field length and width: 71 × 50 mm). Sham control animals (group D, n = 5) were anesthetized but not subjected to irradiation. During the irradiation, the mice in each group were covered with 1 cm tissue equivalent material to achieve a relatively uniform dose distribution throughout the underlying tissue. A single absorbed dose of 2 Gy was administered in each case. The dose variation within the target volume was estimated to be 5%, and the cumulative uncertainty of the radiation dose was estimated to be less than 8%. Animals were injected i.p. with Antisedan (Orion Pharma Animal Health; Sweden) as an antidote to anesthesia. The entire procedure including anesthetization was completed within approximately 45 min and all efforts were made to minimize discomfort or stress for the animals.

### Sample preparation and microarray analysis

Twenty-four hours after irradiation, the animals were killed under anesthesia (Pentobarbitalnatrium vet., i.p.; Apotek Produktion & Laboratorier AB; Sweden) via cardiac puncture and the kidneys, liver, lungs, spleen, and thyroid were excised. The workflow of sample preparation and data processing is illustrated in Fig. 6. Organs were snap-frozen in liquid nitrogen and stored at −80 °C until further sample preparation. The kidney cortex and medulla were dissected on dry ice and treated separately in the analysis. Several milligram of tissue were dissected on dry ice and homogenized using the TissueLyser LT (Qiagen; Hilden, Germany). Total RNA was extracted using the RNeasy Lipid Tissue Mini Kit (Qiagen; Hilden, Germany) according to the manufacturer’s instructions. RNA integrity number (RIN) values were determined with the RNA 6000 Nano LabChip Kit and Agilent 2100 Bioanalyzer (Agilent Technologies; Palo Alto, CA, USA). All RNA samples had a RIN value of at least 6.0. RNA concentration was determined with the ND-1000 Spectrophotometer (NanoDrop Technologies; Wilmington, DE, USA). RNA samples were subjected to Illumina MouseRef-8v2 Whole-Genome Expression BeadChips (Illumina; San Diego, CA, USA) for genome-wide transcriptional analysis. Microarray beadchips were processed at the Swegene Center for Integrative Biology Genomics DNA Microarray Resource Center (SCIBLU; Lund, Sweden). Data preprocessing and quantile normalization were performed with the BioArray Software Environment (BASE, SCIBLU)^36^.

### Microarray data validation

The QPCR assay was used to validate microarray data. Kidney medulla samples from group A and the control group were used for validation. For each sample, cDNA was synthesized from 1 μg total RNA, i.e. from the same RNA eluate committed to microarray analysis. The SuperScript™ III First-Strand Synthesis SuperMix (Invitrogen, Thermo Fisher Scientific; Carlsbad, CA, USA) was used for reverse transcription according to the manufacturer´s protocol. Seven genes that were significantly overexpressed in the microarray data (i.e. *Akr1b3*, *Gjb2*, *Ndufb9*, *Slc11a1*, *Slc38a2*, *Trp53inp1*, *Vbp1*) were selected for QPCR validation. For normalization, three constitutive genes were chosen that showed homogeneous expression across the entire data set (*Cplx1*, *Kcnc4*, and *Slc10a4*). QPCR assays were performed using validated TaqMan^®^ Gene Expression Assays and the TaqMan^®^ Gene Expression Master Mix (Applied Biosystems, Thermo Fisher Scientific; Carlsbad, CA, USA). cDNA samples were run in triplicate for each assay and differential expression was quantified using the ∆∆Ct method.

### Microarray data analysis

The microarray data used in this study have been deposited in NCBI’s Gene Expression Omnibus (http://www.ncbi.nlm.nih.gov/geo/) with accession GEO:GSE66372. Nexus Expression 3.0 (BioDiscovery; El Segundo, CA, USA) was used for data processing and statistical analysis as described elsewhere^37^. Differentially expressed transcripts between irradiated groups and controls were identified according to the Benjamini-Hochberg method (false discovery rate adjusted *p* < 0.01^38^ and with a log_2_ ratio of at least 0.58. Ingenuity Pathway Analysis (IPA, Ingenuity^®^ Systems, www.ingenuity.com; Redwood City, CA) was used to predict downstream effects of transcriptional regulation regarding diseases and biological functions (Fisher’s exact test, *p* < 0.05)^39^. Transcript-associated Gene Ontology (GO, http://www.geneontology.org) terms were enriched for biological processes using Nexus Expression 3.0 (Fisher’s exact test, *p* < 0.05)^40^.

In order to obtain comprehensive profiles of cellular functions that were regulated at the transcriptional level, biological processes were categorized into eight main categories with over 30 subcategories according to ancestor charts in the Gene Ontology database (http://www.geneontology.org) as described previously^27^. The intensity of response was expressed by the percentage of scored vs. filtered transcripts of all biological processes grouped within a category or respective subcategory. Percentages were categorized as very low (<3%), low (3–9%), medium (10–29%), high (30–49%), and very high (≥50%).

Signature gene analysis was used to evaluate the relative impact of direct IR exposure and TH-induced regulation on the overall transcriptomic response. The number of significantly regulated genes in each signature is taken as a relative measure of respective regulatory impact on regulation for each tissue across the different irradiation setups. The IR-associated gene signature was composed of 56 genes adapted from Snyder and Morgan^41^ and Chaudhry^42^. The thyroid hormone-associated gene signature was composed of 61 TH-responding genes (and gene groups encoding multimeric protein) adapted from literature as presented previously^28^. The complete lists of IR-associated and TH-associated signature genes (including information on known RNA expression levels of human homologs) are available as supplementary information (Supplementary Tables S10 and S11, respectively).

## Additional Information

**How to cite this article**: Langen, B. *et al*. Non-targeted transcriptomic effects upon thyroid irradiation: similarity between in-field and out-of-field responses varies with tissue type. *Sci. Rep.* **6**, 30738; doi: 10.1038/srep30738 (2016).

## Supplementary Material

Supplementary Information

## Figures and Tables

**Table 1:** Number of significantly regulated transcripts and genes.

Tissue	Irradiated tissue (group)					
	Thyroid (group A)		Kidneys, liver, lungs, spleen (group B)		Kidneys, liver, lungs, spleen, thyroid (group C)	
Kidney cortex	62 (53)	↑10 (9)	89 (80)	↑33 (32)	84 (78)	↑50 (46)
		↓52 (44)		↓56 (48)		↓34 (32)
Kidney medulla	252 (243)	↑204 (199)	274 (262)	↑228 (221)	259 (238)	↑141 (138)
		↓48 (44)		↓46 (41)		↓118 (100)
Liver	62 (56)	↑25 (21)	36 (33)	↑11 (10)	132 (109)	↑59 (48)
		↓37 (35)		↓25 (23)		↓73 (61)
Lungs	3 (3)	↑3 (3)	70 (62)	↑9 (6)	29 (27)	↑18 (16)
		↓0 (0)		↓61 (56)		↓11 (11)
Spleen	17 (16)	↑3 (3)	133 (121)	↑67 (60)	135 (116)	↑37 (33)
		↓14 (13)		↓66 (61)		↓98 (83)
Thyroid	115 (102)	↑35 (30)	17 (16)	↑10 (9)	25 (24)	↑25 (24)
		↓80 (72)		↓7 (7)		↓0 (0)

Numbers indicate the total number of significantly regulated transcripts in irradiated mice compared with non-irradiated controls.

Number of corresponding genes are shown in parentheses.

Up-arrow indicates upregulation; down-arrow indicates downregulation.
