# Supplementary material for: Non-targeted transcriptomic effects upon thyroid irradiation: similarity between in-field and out-of-field responses varies with tissue type
Source: Sci Rep. 2016 Oct 25;6:30738. doi: 10.1038/srep30738 (PMC5078841; doi:10.1038/srep30738)
Supplement: Supplementary Information [file srep30738-s1.pdf]

Non-targeted transcriptomic effects upon thyroid irradiation: similarity between in-field and out-of-field responses varies with tissue type

Britta Langen, Nils Rudqvist, Johan Spetz, John Swanpalmer, Khalil Helou, Eva Forssell-Aronsson

Supplementary Information

Supplementary Table S1. QPCR validation of microarray data

Supplementary Table S2. Response in ionizing radiation-associated signature genes

Supplementary Table S3. Response in thyroid hormone-responding signature genes

Supplementary Table S4. IPA pathway analysis of diseases and biological functions in the kidney cortex

Supplementary Table S5. IPA pathway analysis of diseases and biological functions in the kidney medulla

Supplementary Table S6. IPA pathway analysis of diseases and biological functions in the liver

Supplementary Table S7. IPA pathway analysis of diseases and biological functions in the lungs

Supplementary Table S8. IPA pathway analysis of diseases and biological functions in the spleen

Supplementary Table S9. IPA pathway analysis of diseases and biological functions in the thyroid

Supplementary Table S10. List of ionizing radiation-associated genes

Supplementary Table S11. List of thyroid hormone-responding genes

Supplementary Figure S1. Transcripts regulated at all irradiation setups

Supplementary Figure S2. Shared transcript regulation among thyroid and other tissues

Supplementary Table S1. QPCR validation of microarray data

Target gene	Differential regulation in the kidney medulla upon thyroid irradiation		Validation
	**QPCR** Thyroid cDNA (log_2)	**Microarray data** Thyroid total RNA (log_2)	
Gjb2	1.00	0.75	Yes
Trp53inp1	1.10	0.75	Yes
Vbp1	0.73	1.17	Yes
Akr1b3	1.32	1.26	Yes
Ndufb9	0.70	1.06	Yes
Slc11a1	0.96	1.17	Yes
Slc38a2	1.52	1.27	Yes

Reference genes were chosen from microarray data that showed lowest overall transcriptional variation (i.e. *Cplx1, Kcnc4* and *Slc10a4*).

Supplementary Table S2. Response in ionizing radiation-associated signature genes

Irradiated tissues:	Thyroid				Kidneys, liver, lungs, spleen				Kidneys, liver, lungs, spleen, thyroid			
Tissue	Gene symbol	Probe ID	Transcript ID	Log_2 ratio	Gene symbol	Probe ID	Transcript ID	Log_2 ratio	Gene symbol	Probe ID	Transcript ID	Log_2 ratio
Kidney cortex					*Ccng1*	ILMN_2500276	ILMN_232357	0.83	*Bax*	ILMN_2907655	ILMN_211762	0.70
					Cdkn1a	ILMN_2634083	ILMN_209664	1.16	*Cdkn1a*	ILMN_2634083	ILMN_209664	1.60
										ILMN_2846775	ILMN_209664	0.73
Kidney medulla	*Gjb2*	ILMN_1227148	ILMN_210347	0.75	*Ccng1*	ILMN_2500276	ILMN_232357	0.71	*Cdkn1a*	ILMN_2634083	ILMN_209664	1.58
	Trp53inp1	ILMN_2971479	ILMN_194391	0.75		ILMN_2702233	ILMN_232357	0.68		ILMN_2846775	ILMN_209664	0.99
						ILMN_2710229	ILMN_232357	0.73	*Gja1*	ILMN_1244291	ILMN_217762	0.79
					Cdkn1a	ILMN_2634083	ILMN_209664	1.00	*Gjb2*	ILMN_1227148	ILMN_210347	0.64
					Gjb2	ILMN_1227148	ILMN_210347	0.70	*Trp53inp1*	ILMN_2506012	ILMN_194391	0.86
					Trp53inp1	ILMN_2971479	ILMN_194391	0.89		ILMN_2971479	ILMN_194391	0.90
Liver	*Gadd45g*	ILMN_2744890	ILMN_222120	-0.73	*Ccnd1*	ILMN_2669793	ILMN_210028	0.61	*Cdkn1a*	ILMN_2634083	ILMN_209664	1.84
		ILMN_2903945	ILMN_222120	-0.85	*Cdkn1a*	ILMN_2634083	ILMN_209664	1.05		ILMN_2846775	ILMN_209664	1.23
					Gadd45g	ILMN_2744890	ILMN_222120	-0.99	*Gadd45a*	ILMN_2742152	ILMN_221926	-0.95
						ILMN_2903945	ILMN_222120	-1.09		ILMN_2947568	ILMN_221926	-0.81
Lungs					*Bax*	ILMN_2907655	ILMN_211762	0.82	*Cdkn1a*	ILMN_2634083	ILMN_209664	1.51
					Cdkn1a	ILMN_2634083	ILMN_209664	1.29		ILMN_2846775	ILMN_209664	1.52
						ILMN_2846775	ILMN_209664	1.36		ILMN_2846776	ILMN_209664	1.07
						ILMN_2846776	ILMN_209664	0.82				
Spleen					*Ccng1*	ILMN_2500276	ILMN_232357	1.11	*Bax*	ILMN_2907655	ILMN_211762	0.66
						ILMN_2702233	ILMN_232357	1.14	*Ccng1*	ILMN_2702233	ILMN_232357	0.95
						ILMN_2710229	ILMN_232357	1.19		ILMN_2710229	ILMN_232357	1.01
					Cdkn1a	ILMN_2634083	ILMN_209664	2.40	*Cdkn1a*	ILMN_2634083	ILMN_209664	2.76
						ILMN_2846775	ILMN_209664	1.51		ILMN_2846775	ILMN_209664	1.83
					Gja1	ILMN_1244291	ILMN_217762	1.01		ILMN_2846776	ILMN_209664	1.23
					Trp53inp1	ILMN_2506012	ILMN_194391	0.96				
						ILMN_2971479	ILMN_194391	0.98				
Thyroid	*Gjb2*	ILMN_1227148	ILMN_210347	1.64								
		ILMN_2999627	ILMN_210347	2.73								

Supplementary Table S3. Response in thyroid hormone-responding signature genes

Irradiated tissues:	Thyroid				Kidneys, liver, lungs, spleen				Kidneys, liver, lungs, spleen, thyroid			
**Tissue**	**Gene symbol**	**Probe ID**	**Transcript ID**	**Log_2 ratio**	**Gene symbol**	**Probe ID**	**Transcript ID**	**Log_2 ratio**	**Gene symbol**	**Probe ID**	**Transcript ID**	**Log_2 ratio**
Kidney cortex					*Cdkn1a*	ILMN_2634083	ILMN_209664	1.16	*Cdkn1a*	ILMN_2634083	ILMN_209664	1.60
					Egf	ILMN_2684104	ILMN_217509	-0.62		ILMN_2846775	ILMN_209664	0.73
					Gsta1	ILMN_2979237	ILMN_196592	-0.68	*Thrsp*	ILMN_1256775	ILMN_209127	0.83
					Thrsp	ILMN_1256775	ILMN_209127	0.90				
Kidney medulla	*G6pc*	ILMN_1245103	ILMN_219085	-0.68	*Cdkn1a*	ILMN_2634083	ILMN_209664	1.00	*Cdkn1a*	ILMN_2634083	ILMN_209664	1.58
	Gsta1	ILMN_2979237	ILMN_196592	0.90	*G6pc*	ILMN_1245103	ILMN_219085	-0.84		ILMN_2846775	ILMN_209664	0.99
	Pck1	ILMN_1213632	ILMN_238641	0.86	*Pck1*	ILMN_1213632	ILMN_238641	0.98	*G6pc*	ILMN_1245103	ILMN_219085	-1.08
					Thrsp	ILMN_1256775	ILMN_209127	-0.69	*Thrsp*	ILMN_1256775	ILMN_209127	-0.83
Liver	*Cyp7a1*	ILMN_2604383	ILMN_210317	0.60	*Ccnd1*	ILMN_2669793	ILMN_210028	0.61	*Cdkn1a*	ILMN_2634083	ILMN_209664	1.84
	Egfr	ILMN_3128725	ILMN_207468	-0.58	*Cdkn1a*	ILMN_2634083	ILMN_209664	1.05		ILMN_2846775	ILMN_209664	1.23
	G6pc	ILMN_1245103	ILMN_219085	-0.71	*Pck1*	ILMN_1213632	ILMN_238641	-0.90	*Thrsp*	ILMN_1256775	ILMN_209127	-0.81
	Gsta1	ILMN_2979237	ILMN_196592	0.96								
	Pck1	ILMN_1213632	ILMN_238641	-1.00								
Lungs					*Cdkn1a*	ILMN_2634083	ILMN_209664	1.29	*Cd44*	ILMN_3114585	ILMN_245439	-0.93
						ILMN_2846775	ILMN_209664	1.36	*Cdkn1a*	ILMN_2634083	ILMN_209664	1.51
						ILMN_2846776	ILMN_209664	0.82		ILMN_2846775	ILMN_209664	1.52
										ILMN_2846776	ILMN_209664	1.07
									Thrsp	ILMN_1256775	ILMN_209127	1.12
Spleen					*Cd44*	ILMN_3114585	ILMN_245439	0.75	*Cdkn1a*	ILMN_2634083	ILMN_209664	2.76
					Cdkn1a	ILMN_2634083	ILMN_209664	2.40		ILMN_2846775	ILMN_209664	1.83
						ILMN_2846775	ILMN_209664	1.51		ILMN_2846776	ILMN_209664	1.23
Thyroid	*Atp2a1*	ILMN_2666864	ILMN_216061	-2.41								

Supplementary Table S4. IPA pathway analysis of diseases and biological functions in the kidney cortex

Kidney cortex			
Irradiated tissues:	Thyroid	Kidneys, liver, lungs, spleen	Kidneys, liver, lungs, spleen, thyroid
**Diseases and Bio Functions**	**Activation z-score**		
Hypertrophy		-1.82	-1.64
quantity of cytokine	1.98		1.40
cell death of liver cells		1.45	1.83
quantity of connective tissue cells		-1.56	-1.64
quantity of amino acids		-1.20	-2.00
consumption of oxygen		-1.98	1.04
transport of molecule	-2.36	0.26	-0.21
ingestion by mice		-1.41	-1.40
quantity of protein in blood	0.78	1.17	0.85
quantity of carbohydrate		-2.71	0.07
proliferation of carcinoma cell lines		-0.80	-1.88
concentration of lipid	-1.96	0.17	-0.51
apoptosis of cardiomyocytes		-1.94	-0.60
concentration of hormone	-0.76	0.78	0.87
Lesion Formation		-2.17	-0.18
cellular homeostasis	0.76		1.58
recruitment of phagocytes			2.34
cell death of connective tissue cells		-0.37	1.97
glucose metabolism disorder	0.59	-0.24	-1.41
mass of epididymal fat			2.24
quantity of steroid hormone			-2.24
secretion of lipid		1.06	-1.17
concentration of D-glucose		-2.07	-0.14
quantity of lymphatic system cells			-2.21
cell death of epithelial cells		-0.88	1.33
colony formation of tumor cell lines		-0.21	-2.00
invasion of cells			2.20
thermogenesis			2.19
insulin resistance		-0.90	-1.12
recruitment of macrophages			2.00
concentration of corticosterone			-2.00
concentration of triacylglycerol	-1.49	-0.45	0.05
body temperature			-1.98
metastasis of tumor cell lines			1.98
metastasis of cells			1.97
cell death of lymphatic system cells			1.97
localization of protein		-1.97	
phagocytosis of phagocytes			1.95
phagocytosis of myeloid cells			1.95
energy expenditure		-1.47	0.45
immune response of leukocytes			1.88
quantity of steroid			-1.80
invasion of tumor cell lines			1.79
apoptosis of muscle cells		-1.78	

obesity	0.16		1.60
quantity of glycogen			1.73
secretion of molecule		0.92	-0.81
quantity of tumor cell lines		-1.72	
concentration of fatty acid	-1.17	0.02	0.53
cell death of endothelial cells		0.85	0.85
mass of fat pad	1.41	-0.16	0.13
metastasis			1.69
advanced malignant tumor			1.69
atherosclerosis		-0.82	0.83
feeding		-0.58	-1.07
quantity of reactive oxygen species		-1.11	-0.50
proliferation of smooth muscle cells		-1.60	
organismal death			-1.56
proliferation of fibroblasts			-1.52
hypertrophy of heart		-1.52	
necrosis of tumor		-0.94	-0.57
cell death of tumor cells		-0.94	-0.57
recruitment of leukocytes			1.51
accumulation of lipid		1.38	0.12
apoptosis of tumor cell lines			-1.49
recruitment of neutrophils			1.46
cell viability			1.45
proliferation of lung cancer cell lines			-1.45
concentration of sterol			-1.44
Nephritis			-1.41
resorption of bone			-1.40
insulin sensitivity			-1.39
quantity of bone marrow cells			-1.39
necrosis of epithelial tissue			1.36
hepatic steatosis		0.64	-0.71
Edema			-1.34
lung tumor			1.34
thermoregulation			1.33
disorder of artery		-0.19	1.14
uptake of lipid		1.00	-0.33
quantity of adipose tissue	0.56	0.36	0.40
apoptosis of epithelial cells		-0.94	0.37
activation of myeloid cells			1.30
cell death of brain cells			1.29
invasion of tumor cells			1.29
accumulation of myeloid cells			1.28
size of body			1.26
cell death of muscle cells			-1.24
apoptosis of myeloid cells			1.24
chemotaxis of myeloid cells			1.21
chemotaxis of phagocytes			1.21
size of bone			-1.21
necrosis of liver			1.20
proliferation of tumor cell lines			-1.20
inflammation of organ	0.30		-0.90
synthesis of nitric oxide			1.20
differentiation of leukocytes			1.20
apoptosis of heart		-1.19	
cell death of thymocytes			1.19

synthesis of lipid	0.28	-0.76	0.13
apoptosis of connective tissue cells			1.17
hypoglycemia			-1.17
differentiation of connective tissue cells			1.16
cell death of fibroblasts		-1.15	
cytostasis of tumor cell lines			1.15
vascular lesion			1.15
apoptosis of B-lymphocyte derived cell lines			1.13
cell death of hematopoietic cell lines			1.13
quantity of white adipose tissue			1.13
apoptosis of lymphoid organ			1.13
activation of phagocytes			1.12
tubulation of endothelial cells		-1.11	
cell viability of breast cancer cell lines		-1.10	
bone mineral density			-1.09
accumulation of triacylglycerol		1.09	
Infarction			-1.08
size of infarct			-1.08
formation of reactive oxygen species			1.07
size of adipocytes			1.07
differentiation of B lymphocytes			1.07
mass of kidney		-1.07	
activation of neutrophils			1.07
mass of inguinal fat pad			-1.07
proliferation of epithelial cells			1.05
efflux of cholesterol		1.05	
growth of tumor		0.39	0.66
inflammation of body region	0.20		-0.84
formation of kidney			-1.04
cell death of immune cells			1.03
fatty acid metabolism		-0.18	0.83
necrosis of renal tubule			-1.00
differentiation of fibroblast cell lines		1.00	
migration of carcinoma cell lines			-1.00
apoptosis of brain cells			1.00
hydronephrosis		0.98	
quantity of leukocytes			-0.97
apoptosis of lung cancer cell lines			-0.97
development of abdomen			-0.97
cell death of cardiomyocytes			-0.96
quantity of bone cells			-0.94
cell death of cortical neurons			0.93
adhesion of neutrophils			0.93
neuronal cell death			0.92
apoptosis of leukocytes			0.91
growth of epithelial tissue			0.91
cell death of myeloid cells			0.91
accumulation of cells			0.88
apoptosis of phagocytes			0.87
occlusion of artery		-0.82	
experimental autoimmune encephalomyelitis	0.81		
fertility	-0.15		-0.65
urination disorder		-0.66	-0.14
quantity of leptin in blood			0.79
neoplasia of cells			0.78

secretion of steroid			-0.76
concentration of ATP			-0.76
polyuria		-0.76	
cell death of leukocyte cell lines			0.75
quantity of insulin in blood	-0.25	0.00	0.48
neoplasia of tumor cell lines			0.73
synthesis of reactive oxygen species			0.73
lung cancer			0.71
injury of cells			0.71
mass of adipose tissue			0.70
cell movement of leukocytes			-0.70
proliferation of epithelial cell lines			0.69
apoptosis of hepatoma cell lines		-0.69	
survival of organism			-0.69
atherosclerotic lesion			0.69
homing of cells			0.67
inflammatory response			0.65
activation of leukocytes			0.65
tumorigenesis of cells			-0.64
Parasitic Infection	-0.64		
differentiation of blood cells			0.63
proliferation of hematopoietic progenitor cells			-0.62
cell viability of cancer cells			-0.62
endothelial cell development			0.61
quantity of B lymphocytes			-0.60
synthesis of fatty acid			-0.60
hyperglycemia		0.59	
apoptosis of hematopoietic progenitor cells			0.59
vascular disease		-0.17	0.42
benign neoplasia			-0.58
weight gain		0.58	
infection of mammalia	-0.57		
formation of blood cells			-0.57
size of lesion			-0.57
apoptosis of cortical neurons			0.57
oxidation of fatty acid		0.56	
cell death of phagocytes			0.56
tumorigenesis of epithelial neoplasm			0.55
synthesis of steroid			-0.55
cell movement of neutrophils			-0.54
generation of reactive oxygen species			0.53
stress response of cells			0.51
cell movement			-0.51
autophagy of cells			-0.50
proliferation of connective tissue cells			-0.50
apoptosis of macrophages			0.49
injury of liver		-0.13	0.35
cell movement of macrophages			0.48
behavior			-0.48
development of genitourinary system			-0.48
apoptosis of colon cancer cell lines		-0.48	
apoptosis of tumor cells		-0.47	
cell death of carcinoma cell lines			0.45
activation of macrophages			0.45
activation of antigen presenting cells			0.45
apoptosis of endothelial cells			0.45

abnormal morphology of abdomen			-0.45
lean body mass			-0.45
transmembrane potential of mitochondria			0.45
homing of leukocytes			0.43
cell death of antigen presenting cells			0.43
maturation of cells			-0.43
development of body trunk			-0.43
proliferation of blood cells			-0.41
adhesion of immune cells			0.40
apoptosis of cancer cells		-0.39	
cell death of lung cancer cell lines			-0.39
glucose tolerance			-0.37
vasculogenesis			0.36
apoptosis of breast cancer cell lines			-0.36
steroid metabolism			0.35
inflammation of liver			-0.35
concentration of cholesterol			-0.34
cell movement of tumor cell lines			0.32
quantity of granulocytes			-0.32
infiltration by neutrophils			-0.31
cell death of cancer cells			-0.31
activation of cells			0.30
migration of muscle cells			0.29
accumulation of granulocytes			0.29
formation of lymphatic system component			-0.28
development of lymphatic system component			-0.28
size of cells	-0.28		
development of epithelial tissue			0.26
damage of liver		-0.13	-0.13
lipolysis			0.25
mass of organism	-0.25		
cell death			-0.24
formation of leukocytes			-0.24
infiltration of macrophages			0.24
differentiation of muscle			-0.24
metabolism of carbohydrate		-0.24	
Fibrosis			0.24
production of reactive oxygen species			0.23
stress response of tumor cell lines			-0.22
concentration of cholesterol ester		-0.20	
malignant neoplasm of thorax			0.20
apoptosis of prostate cancer cell lines		0.17	
proliferation of cells			-0.16
differentiation of myoblasts			0.15
delayed hypersensitive reaction			0.15
proliferation of hepatoma cell lines		-0.15	0.00
apoptosis of epithelial cell lines		-0.15	
oxidation of lipid		0.14	
differentiation of connective tissue			0.14
cell movement of phagocytes			-0.14
development of tumor			0.13
quantity of monosaccharide			0.13
leukocyte migration			-0.13
differentiation of muscle cells			0.13
synthesis of carbohydrate		0.13	
cell death of macrophages			0.11

apoptosis of hepatocytes		0.11	
migration of cells			0.11
differentiation of epithelial tissue		0.11	
proliferation of mononuclear leukocytes			0.10
necrosis			-0.10
apoptosis			0.09
quantity of myeloid cells	0.07		
proliferation of endothelial cells			-0.06
uptake of carbohydrate			-0.06
proliferation of tumor cells			0.05
formation of cells			0.04
migration of smooth muscle cells			-0.04
cell movement of myeloid cells			-0.03
quantity of brown adipose tissue			0.02
differentiation of adipocytes			-0.02
infiltration of leukocytes			0.01

Supplementary Table S5. IPA pathway analysis of diseases and biological functions in the kidney medulla

Kidney medulla			
Irradiated tissues:	Thyroid	Kidneys, liver, lungs, spleen	Kidneys, liver, lungs, spleen, thyroid
**Diseases and Bio Functions**	**Activation z-score**		
organismal death	-5.40	-4.74	-3.13
metabolism of reactive oxygen species	2.42	2.10	2.93
Movement Disorders	-2.39	-2.77	-1.46
invasion of tumor cell lines	1.32	1.81	3.40
proliferation of tumor cell lines	2.30	1.14	2.69
acute inflammation	1.98	1.98	1.98
growth of organism	2.18	1.71	1.86
synthesis of carbohydrate	2.78	2.56	
proliferation of cells	1.92	1.35	1.69
invasion of cells		1.43	3.41
growth of tumor	1.32	1.76	1.63
growth of malignant tumor	1.40	1.31	1.83
invasion of breast cancer cell lines		1.38	3.05
synthesis of reactive oxygen species		1.73	2.70
cell death of neuroblastoma cell lines		-1.70	-2.62
migration of vascular smooth muscle cells	1.34	1.34	1.63
quantity of anion	1.34	1.34	1.46
adhesion of breast cancer cell lines	2.00	2.00	
inflammatory response		2.66	1.31
migration of muscle cells		1.72	2.16
cellular homeostasis		1.91	1.77
apoptosis of muscle cells	-0.28	-1.91	1.46
migration of cells		1.60	2.01
cell death of muscle cells	-0.10	-1.75	1.67
modification of reactive oxygen species	1.71	1.71	
thymic lymphoma	-1.71	-1.71	
polyuria		-1.43	-1.98
metastasis		1.00	2.41
advanced malignant tumor		1.00	2.41
necrosis of renal tubule		-2.00	-1.34
proliferation of kidney cells		-1.91	1.41
apoptosis of neuroblastoma cell lines		-1.05	-2.24
transport of molecule		2.79	0.48
necrosis of epithelial tissue		-2.84	-0.36
homeostasis of anion	1.00	1.00	1.20
cell movement		1.47	1.67
cell death of tumor cell lines		-1.68	-1.35
necrosis	-0.93	-1.44	0.65
migration of tumor cell lines			2.81
disorder of artery			2.78
necrosis of tumor		-1.01	-1.73
cell death of tumor cells		-1.01	-1.73
occlusion of artery		0.33	2.41
cell death of epithelial cells		-2.51	-0.20

adhesion of tumor cell lines			2.63
degeneration of cells	-1.35	-1.26	
lung tumor	0.39	0.34	1.71
migration of carcinoma cell lines			2.43
atherosclerosis			2.41
cell movement of tumor cell lines			2.40
development of genitourinary system		1.28	1.11
Parasitic Infection	-2.39		
apoptosis of epithelial cells		-2.39	
experimentally-induced arthritis	1.15		1.24
blood pressure		0.86	1.52
cell movement of muscle cells			2.38
apoptosis of fibroblast cell lines		2.37	
synthesis of fatty acid			2.36
urination disorder	-1.01	-1.24	0.10
weight loss		-2.35	
quantity of osteoclasts			-2.32
apoptosis of macrophages		-1.70	-0.61
quantity of connective tissue cells		0.16	-2.13
proliferation of cancer cells	0.45	0.62	1.21
growth of embryo	1.07		1.20
cell death of fibroblast cell lines		2.24	
transport of metal			2.22
cell death of cardiomyocytes		-1.19	1.01
cell movement of smooth muscle cells			2.19
cytopenia	-0.90	-1.25	
differentiation of epithelial cells		2.14	
vascular disease		0.27	1.86
cell death of heart cells		-1.45	0.68
infiltration by neutrophils		0.86	1.22
invasion of colon cancer cell lines	0.85	1.23	
oxidation of lipid		2.07	
adipogenesis		1.91	-0.13
cytolysis	1.14	0.90	
quantity of white adipose tissue	0.90	-0.38	-0.76
cell death of endothelial cells		-1.46	-0.55
transport of metal ion			2.00
exocytosis by cells			2.00
quantity of IL-1b in blood	-1.00	-1.00	
differentiation of epithelial tissue		2.00	
latency of tumor		1.99	
apoptosis of kidney cells		-1.98	
invasion of tumor			1.98
malignant neoplasm of thorax	-0.60	-0.65	0.73
clearance of lipid		1.97	
lack of mesoderm	-1.96		
quantity of amino acids	-0.74	-0.40	-0.82
cell movement of blood cells		0.87	1.08
apoptosis of keratinocyte cancer cell lines		-1.95	
migration of smooth muscle cells			1.95
Growth Failure			-1.94
aggregation of tumor cell lines		1.92	
mass of fat pad	0.13	-0.89	-0.89
cell movement of vascular smooth muscle cells			1.91
quantity of adipose tissue	0.20	-1.12	-0.57

quantity of connective tissue	0.20	-1.12	-0.57
quantity of bone cells			-1.88
glucose metabolism disorder		-1.86	
concentration of sterol		-1.64	-0.17
cell death of connective tissue cells		1.79	
lean body mass		1.34	0.45
invasion of carcinoma cell lines	1.74		
concentration of lipid		-1.59	-0.15
quantity of IL-6 in blood		0.39	1.34
thickness of cortical bone	1.71		
growth of renal glomerulus			1.71
thymus cancer		-1.71	
concentration of cholesterol		-1.70	
cell viability of breast cancer cell lines		0.20	1.49
Organ Degeneration	-1.68		
lung cancer	0.39	-0.08	1.19
cell movement of myeloid cells		0.64	1.01
Fibrosis	-1.16	-0.48	
survival of organism		1.64	
body temperature	1.61		
fatty acid metabolism			1.56
cell movement of neutrophils		0.90	0.63
sarcoma	-0.52	-0.94	-0.06
synthesis of lipid		0.67	0.86
cell movement of colon cancer cell lines		1.52	
tumorigenesis of epithelial neoplasm			1.51
concentration of levothyroxine	-0.75		-0.75
development of blood vessel			1.49
oxidation of fatty acid		1.48	
formation of mononuclear leukocytes		0.65	0.82
Neurodegeneration		-0.94	-0.53
apoptosis of tumor cell lines			-1.46
quantity of steroid		-0.86	-0.59
apoptosis of stem cells		-1.45	
thermoregulation	1.45		
migration of endothelial cells			1.42
formation of leukocytes		1.00	0.41
apoptosis of lung cancer cell lines			-1.41
development of cardiovascular system			1.41
senescence of tumor cell lines	-1.18	0.20	
quantity of filaments		-1.33	
quantity of Na+		0.76	-0.55
soft tissue sarcoma		-1.09	-0.22
resorption of bone			-1.30
skin tumor			-1.29
development of reproductive system			1.29
cell movement of phagocytes		0.57	0.70
cell death of macrophages		-1.25	
vasculogenesis			1.24
neurodegeneration of hair cells		1.24	
apoptosis of epithelial cell lines		-1.23	
homing of blood cells			1.23
binding of cells		1.22	
generation of reactive oxygen species		1.22	
osmolality of urine		1.21	

G1 phase		1.20	
proliferation of cervical cancer cell lines			1.20
apoptosis of myeloid cells		-0.92	-0.27
concentration of triacylglycerol		-1.14	0.05
systemic autoimmune syndrome		1.15	
apoptosis of phagocytes		-0.88	-0.27
proliferation of connective tissue cells		0.67	0.47
tumorigenesis of benign tumor		0.58	0.55
clearance of virus		1.12	
reduction of carbohydrate		1.12	
aggregation of cells			1.12
tumorigenesis of lymphoma		-1.11	
formation of hematopoietic progenitor cells			1.11
bleeding of tissue	-1.09		
differentiation of erythroid progenitor cells			-1.07
Thrombosis			-1.07
scattering of cells	-1.07		
synthesis of D-hexose		1.07	
mobilization of blood cells			1.07
abnormal morphology of kidney		1.07	
differentiation of red blood cells			-1.07
apoptosis of cardiomyocytes			1.07
formation of kidney		0.81	0.25
cell movement of antigen presenting cells		1.06	
formation of blood cells		1.00	0.06
degeneration of nervous system		-0.85	-0.20
G1/S phase transition		1.04	
steroid metabolism			1.04
epithelial-mesenchymal transition of tumor cell lines	0.34	0.68	
apoptosis		0.14	0.88
homing of leukocytes			1.00
contraction of heart			1.00
translation of protein	1.00		
transcription of mRNA			1.00
urological cancer		-1.00	0.00
quantity of chloride		1.00	0.00
cell movement of macrophages		1.00	
cell death	0.11	-0.10	0.79
bone mineral density	0.63		-0.36
degeneration of neurons		-0.98	
apoptosis of lymphoid organ			0.98
dissemination of cells		0.97	
degeneration of cochlea		0.97	
proliferation of smooth muscle cells		0.50	0.46
neoplasia of cells			0.95
stress response of cells			0.93
incidence of head and neck tumor	-0.93		
infiltration of macrophages		0.92	
homing of cells			0.90
invasion of tissue		0.90	
proteinuria		-0.30	0.59
growth of epithelial tissue			0.87
contact growth inhibition of tumor cell lines			0.87
ion homeostasis of cells		0.77	0.09
colony formation of carcinoma cell lines			0.85

concentration of fatty acid		-0.85	
apoptosis of carcinoma cell lines			-0.84
production of superoxide		-0.82	
cell death of renal tubule			-0.82
cell movement of leukocytes		0.82	
Lymphoid Cancer and Tumors		-0.82	
apoptosis of colon cancer cell lines		-0.36	-0.46
diabetes mellitus		-0.78	
proliferation of tumor cells		0.53	-0.25
transport of inorganic cation			0.77
neurodegeneration of nerves		0.76	
coordination	0.31	0.44	
fragmentation of DNA		0.74	
renal impairment		-0.73	
failure of kidney		-0.73	
leukocyte migration		0.72	
cellular degradation	-0.06	-0.65	
apoptosis of heart cells			0.69
hypercalcemia			0.69
quantity of brown adipose tissue		-0.69	
chemotaxis of myeloid cells			0.68
chemotaxis of phagocytes			0.68
Hypertrophy		-0.09	0.59
quantity of epithelial cells			-0.65
cell death of myeloid cells		-0.64	
cell death of phagocytes		-0.61	
Lesion Formation	0.08	-0.48	-0.04
endothelial cell development			0.58
cell movement of prostate cancer cell lines		0.57	
concentration of D-glucose		-0.56	
infiltration of phagocytes		0.56	
adenoma		-0.45	0.12
injury of cells			0.56
synthesis of sterol			-0.55
metabolism of hydrogen peroxide		-0.55	
lymphoid cancer		-0.55	
obesity			0.55
concentration of hormone			-0.54
cell movement of fibroblast cell lines			0.53
migration of fibroblast cell lines			0.53
size of lesion	-0.18	-0.28	-0.04
proliferation of carcinoma cell lines		0.00	0.50
adhesion of neutrophils			0.49
growth of carcinoma		0.49	
proliferation of muscle cells			0.48
quantity of carbohydrate		-0.47	
quantity of vitamin			-0.47
damage of kidney			0.46
differentiation of hematopoietic progenitor cells			0.46
hematologic cancer		-0.46	
damage of bone	-0.22	0.12	0.12
injury of liver			0.45
size of infarct	-0.44		
interphase of tumor cell lines		0.42	
cell movement of fibroblasts			0.42
proliferation of hematopoietic progenitor cells			-0.40

production of reactive oxygen species		0.40	
carcinoma in lung		0.39	
adipogenesis of fibroblast cell lines	-0.39		
development of body trunk			0.39
progression of tumor		0.38	
apoptosis of B-lymphocyte derived cell lines			0.37
cell death of cervical cancer cell lines			0.37
benign neoplasia		0.21	0.16
metabolism of hormone			0.36
synthesis of hormone			0.36
apoptosis of brain cells			0.35
metabolism of nucleotide			0.34
metabolism of nucleic acid component or derivative			0.34
proliferation of endothelial cells			0.34
quantity of tumor	-0.12	-0.10	-0.12
abnormal morphology of abdomen		-0.17	-0.15
development of cardiovascular tissue			0.32
development of endothelial tissue			0.32
infiltration of blood cells		0.31	
differentiation of cells	-0.29		
synthesis of steroid			-0.29
growth of connective tissue		0.20	-0.10
head and neck cancer			-0.28
proliferation of myeloid cells			0.24
hearing loss			-0.24
proliferation of colon cancer cell lines		0.24	
tumorigenesis of cells			-0.24
proliferation of vascular smooth muscle cells			-0.24
proliferation of fibroblasts			0.21
colony formation of tumor cell lines			-0.21
development of abdomen			-0.20
fibrosis of kidney		-0.20	
glomerulosclerosis		-0.19	
proliferation of brain cancer cell lines			-0.17
quantity of stem cells			-0.15
quantity of actin filaments		-0.15	
quantity of actin stress fibers		-0.15	
uptake of anion			-0.15
infiltration of leukocytes		0.14	
damage of lung			-0.13
re-entry into cell cycle progression		0.13	
anoikis		-0.12	
proliferation of vascular endothelial cells			0.11
apoptosis of central nervous system cells			-0.11
apoptosis of tumor cells		-0.11	
proliferation of dermal cells		0.10	
adhesion of endothelial cell lines			0.09
respiration of mitochondria	0.08		
transport of ion			0.08
development of epithelial tissue			0.08
development of tumor		-0.07	
mass of kidney		0.00	0.07
Lung Cancer and Tumors		-0.07	
steroidogenesis of hormone			0.06
concentration of glutathione		-0.05	

Supplementary Table S6. IPA pathway analysis of diseases and biological functions in the liver

Liver			
Irradiated tissues:	Thyroid	Kidneys, liver, lungs, spleen	Kidneys, liver, lungs, spleen, thyroid
**Diseases and Bio Functions**	**Activation z-score**		
damage of liver	1.99	1.99	1.99
mass of adipose tissue	-1.98	-2.14	0.51
quantity of steroid	1.05	1.47	1.54
quantity of carbohydrate	-1.24	-2.20	-0.52
synthesis of lipid	-1.42	-1.93	0.57
oxidation of fatty acid	2.00		1.35
concentration of fatty acid	1.35		1.80
apoptosis of epithelial cells	1.00	1.91	
quantity of adipose tissue		-2.13	0.70
oxidation of lipid	1.83		0.82
apoptosis of tumor cell lines	-0.61	-1.94	
concentration of lipid	0.96	0.03	1.46
invasion of cells		1.41	0.95
conversion of lipid	-0.91		-1.33
concentration of D-glucose	-1.39		0.84
development of genitourinary system	0.24	-1.98	
development of abdomen		-2.21	
lean body mass		-1.07	-1.07
mass of fat pad		-1.95	-0.16
feeding		-1.07	-1.00
morbidity or mortality			1.98
morphology of cells	-1.97		
Shock Response			1.95
cell movement of myeloid cells		-0.13	1.71
behavior		-0.39	-1.35
concentration of hormone	-0.76		0.98
binding of tumor cell lines			1.73
development of body trunk		-1.73	
weight gain			-1.72
accumulation of lipid	-1.23	-0.39	0.08
cell viability of breast cancer cell lines		-1.69	
proliferation of connective tissue cells		-1.64	
fatty acid metabolism	-0.78		-0.82
size of body	-1.41	-0.17	
urination disorder		-1.10	-0.47
inflammation of organ	1.09		0.41
necrosis of epithelial tissue		1.47	
synthesis of steroid	-0.86		0.59
necrosis of liver	1.43		
weight loss			1.43
proliferation of fibroblasts		-1.42	
adhesion of blood cells		-1.41	
accumulation of cells		-1.38	
interphase		1.37	

concentration of triacylglycerol	0.89	0.07	0.40
apoptosis of prostate cancer cell lines	-1.30		
organismal death			1.29
activation of cells		-1.25	
metabolism of hormone	-1.19		
synthesis of carbohydrate		-1.18	
inflammation of body region	0.70		0.47
size of infarct		-1.17	
proliferation of brain cancer cell lines			-1.15
proliferation of cancer cells		1.13	
formation of filaments		-1.13	
aggregation of cells		1.11	
release of fatty acid			-1.10
invasion of tumor cell lines		1.09	
mass of liver	1.07		
size of connective tissue cells			1.07
quantity of monosaccharide	-1.07		
generation of reactive oxygen species		-1.03	
steroid metabolism	-0.31		-0.71
quantity of protein in blood			1.02
lipolysis			-1.01
proliferation of hematopoietic progenitor cells		-1.00	
accumulation of cholesterol			-1.00
proliferation of vascular endothelial cells		-1.00	
size of lesion			-1.00
energy expenditure		1.00	
hepatic steatosis	0.38	0.00	0.61
size of adipocytes		-0.96	
metabolism of eicosanoid			-0.95
ingestion by mice			-0.95
inflammatory response		-0.95	
metabolism of terpenoid	-0.73		-0.17
concentration of cholesterol	0.34	-0.24	0.32
apoptosis	-0.54	-0.32	
Growth Failure	-0.86		
inflammation of liver	-0.85		
quantity of hdl cholesterol in blood			0.85
tumorigenesis of epithelial neoplasm		0.83	
concentration of acylglycerol	0.83		
differentiation of tumor cell lines	-0.83		
colony formation of tumor cell lines	0.80		
hyperactive behavior			-0.76
tumorigenesis of malignant tumor		0.76	
lipolysis of cells			-0.75
flux of cholesterol			-0.74
vascular disease		0.07	0.66
metabolism of carbohydrate	-0.73		
proliferation of prostate cancer cell lines	-0.17	0.55	
glucose tolerance			-0.71
proliferation of cells	-0.45	-0.13	-0.06
release of lipid			-0.62
size of cells		-0.62	
binding of DNA		-0.60	
glucose metabolism disorder	0.26	-0.10	-0.22
thermoregulation			0.58

benign neoplasia	-0.58	0.00	
inflammation of lung	0.55		
proliferation of fibroblast cell lines		-0.52	
synthesis of sterol			0.51
cell death of epithelial cells	0.48		
cell death	-0.48		
synthesis of fatty acid			0.48
hyperglycemia			0.48
proliferation of tumor cells		0.46	
uptake of monosaccharide	-0.46		
quantity of ketone body			0.45
hydroxylation of lipid	0.20		-0.22
differentiation of cells		-0.42	
synthesis of reactive oxygen species			-0.41
metabolism of reactive oxygen species			-0.41
growth of epithelial tissue		0.40	
development of tumor		-0.38	
disorder of artery			0.35
expansion of cells		0.34	
inflammation of body cavity	0.33		
formation of cellular protrusions		-0.33	
growth of organism	-0.32		
migration of cells			0.29
consumption of oxygen			-0.28
development of reproductive system	0.28		
hyperplasia	-0.28		
Edema		-0.25	
arteriosclerosis			0.25
development of epithelial tissue		0.24	
accumulation of triacylglycerol			-0.22
perinatal death			0.21
Fibrosis	-0.20		
efflux of lipid			0.18
proliferation of breast cancer cell lines		-0.18	
Lesion Formation			-0.17
efflux of cholesterol		-0.16	
contractility of muscle			0.15
quantity of leptin in blood			0.15
leukocyte migration		-0.15	
insulin sensitivity			-0.14
flux of lipid			-0.13
proliferation of endothelial cells		-0.13	
incidence of tumor		0.10	
Nephritis			0.08
mitosis		0.08	
obesity			-0.06
necrosis	0.03		
cellular homeostasis		-0.03	

Supplementary Table S7. IPA pathway analysis of diseases and biological functions in the lungs

Lungs			
Irradiated tissues:	Thyroid	Kidneys, liver, lungs, spleen	Kidneys, liver, lungs, spleen, thyroid
**Diseases and Bio Functions**	**Activation z-score**		
hypertrophy of heart		1.57	-1.98
inflammation of organ		-1.48	-1.84
activation of myeloid cells		-1.39	-1.73
inflammation of body region		-1.21	-1.80
leukocyte migration		-0.96	-1.20
inflammatory response		-0.96	-1.10
vasculogenesis		-0.06	-1.97
apoptosis of hematopoietic progenitor cells			-2.00
mass of heart		1.98	
vascular lesion			-1.98
proliferation of carcinoma cell lines			-1.98
experimental autoimmune encephalomyelitis			-1.98
activation of leukocytes		-1.98	
thermogenesis			1.96
synthesis of DNA			-1.96
Lesion Formation			-1.94
proliferation of smooth muscle cells			-1.93
glucose metabolism disorder			-1.91
activation of phagocytes			-1.91
proliferation of endothelial cells			-1.89
activation of lymphocytes			-1.82
angiogenesis		0.15	-1.61
differentiation of connective tissue cells			1.74
ingestion by mice			-1.73
extension of neurites		1.73	
resorption of bone			-1.73
remodeling of bone			-1.73
accumulation of cells			-1.71
proliferation of lymphocytes			-1.67
hepatic steatosis			-1.66
vascular disease			-1.61
activation of T lymphocytes			-1.56
proliferation of T lymphocytes			-1.54
chemotaxis of cells		-1.52	
Bacterial Infection		1.52	
size of body			1.52
quantity of Ca2+		-1.49	
migration of endothelial cells			1.41
concentration of lipid			1.40
cell movement		-1.38	
concentration of fatty acid			1.38
contractility of muscle		-1.35	
organismal death			-1.31
atherosclerosis			-1.29

development of endothelial tissue		-1.28
injury of liver		1.23
concentration of triacylglycerol		1.22
cell death of antigen presenting cells	-1.21	
adhesion of endothelial cells		-1.21
Hypertrophy	1.20	
obesity		1.18
quantity of connective tissue cells		-1.16
attachment of cells		-1.15
metabolism of carbohydrate		-1.11
secretion of molecule	1.11	
cell movement of fibroblasts		-1.09
organization of cytoskeleton		1.09
recruitment of leukocytes		-1.07
colony formation of tumor cell lines		-1.01
glucose tolerance		1.01
apoptosis of phagocytes	-1.00	
apoptosis of myeloid cells	-1.00	
activation of macrophages	-1.00	
energy expenditure		-1.00
behavior		-1.00
formation of filaments		1.00
thermoregulation		1.00
quantity of metal	-0.98	
differentiation of adipocytes		0.97
proliferation of connective tissue cells		-0.97
contractility of heart	-0.96	
contractility of cardiac muscle	-0.96	
apoptosis of lymphocytes		-0.95
cell death of connective tissue cells		-0.93
quantity of leukocytes	-0.92	
urination disorder		-0.91
synthesis of fatty acid		-0.88
development of genitourinary system		-0.85
synthesis of lipid		0.84
binding of cells	0.84	
quantity of reactive oxygen species	0.24	0.60
killing of cells	-0.83	
migration of cells	-0.82	
development of epithelial tissue		-0.78
synthesis of carbohydrate		-0.76
quantity of protein in blood		-0.72
proliferation of fibroblasts		-0.71
cell movement of phagocytes	-0.71	
blood pressure		-0.71
lung cancer		-0.69
apoptosis of connective tissue cells		-0.69
damage of liver		0.66
cell movement of leukocytes	-0.65	
cell death of epithelial cells		0.64
apoptosis of fibroblasts	0.62	
generation of reactive oxygen species	0.59	
quantity of cells	-0.57	
respiratory quotient		0.55
cell movement of macrophages		-0.55

production of reactive oxygen species	-0.39	0.11
quantity of cytokine		-0.51
concentration of cholesterol		0.49
concentration of D-glucose		-0.48
heart rate	-0.12	0.34
quantity of steroid		-0.45
weight loss		-0.45
concentration of hormone		0.40
size of bone		-0.39
synthesis of reactive oxygen species	-0.34	
morbidity or mortality	0.15	0.15
incidence of tumor		-0.30
binding of DNA		0.27
consumption of oxygen		0.20
mass of fat pad		-0.17
accumulation of triacylglycerol		-0.16
cell movement of myeloid cells	0.16	
proliferation of hepatoma cell lines		-0.15
development of tumor		-0.15
quantity of leptin in blood		0.15
release of metal	-0.14	
insulin sensitivity		-0.13
production of superoxide		-0.11
quantity of bone cells		-0.11
aggregation of cells		-0.09
invasion of breast cancer cell lines		-0.08
apoptosis		0.08
invasion of cells	0.04	
quantity of adipose tissue		-0.02
invasion of tumor cell lines		0.01

Supplementary Table S8. IPA pathway analysis of diseases and biological functions in the spleen

Spleen			
Irradiated tissues:	Thyroid	Kidneys, liver, lungs, spleen	Kidneys, liver, lungs, spleen, thyroid
**Diseases and Bio Functions**	**Activation z-score**		
activation of leukocytes		-2.54	-2.34
inflammatory response		-3.39	-1.47
activation of cells	-0.93	-2.10	-1.63
quantity of blood cells		0.99	-3.38
adhesion of immune cells		2.52	-1.82
quantity of lymphocytes			-4.26
activation of lymphocytes		-1.44	-2.77
homing of leukocytes		-2.36	-1.84
Rheumatic Disease		-2.20	-1.85
cell movement of phagocytes		-2.20	-1.75
arthritis		-1.96	-1.98
quantity of mononuclear leukocytes			-3.93
quantity of cells		1.48	-2.43
cell movement of neutrophils		-2.47	-1.31
chemotaxis of myeloid cells		-2.71	-1.05
killing of cells		-1.97	-1.68
chemotaxis of phagocytes		-2.52	-1.12
quantity of hematopoietic progenitor cells		1.66	-1.97
phagocytosis		1.67	-1.92
Bacterial Infection		0.66	2.93
chemotaxis of neutrophils		-2.17	-1.39
quantity of B lymphocytes			-3.51
immune response of leukocytes		-0.68	-2.79
cell movement of mononuclear leukocytes		-0.94	-2.51
cell movement of myeloid cells		-2.06	-1.33
homing of mononuclear leukocytes		-1.73	-1.62
cell movement		-0.94	-2.38
quantity of leukocytes			-3.30
quantity of IgG1			-3.24
phagocytosis of cells		1.43	-1.71
engulfment of leukocytes		0.98	-2.15
immune response of cells		1.15	-1.97
cell movement of granulocytes		-1.90	-1.21
infection of mammalia		0.35	2.76
cell movement of leukocytes		-1.09	-1.89
quantity of T lymphocytes			-2.98
morphology of leukocytes			2.95
morphology of blood cells			2.95
cell death of cancer cells		-2.07	-0.87
quantity of IgG			-2.91
activation of myeloid cells		-2.87	
maturation of blood cells		1.08	-1.73
morphology of lymphocytes			2.79
lack of lymphocytes			2.79

leukopenia		2.79
cytopenia		2.79
binding of leukocytes	2.73	
hemostasis	-2.73	
activation of blood cells	-2.71	
activation of phagocytes	-2.70	
cell movement of antigen presenting cells	0.65	-2.03
leukocyte migration	-0.43	-2.21
migration of cells	-0.64	-2.00
proliferation of hematopoietic progenitor cells		-2.61
engulfment of phagocytes	0.63	-1.94
morphology of cells		2.55
cell movement of lymphocytes		-2.52
activation of macrophages	-2.47	
morphology of B lymphocytes		2.41
quantity of lymphatic system component		-2.41
lack of B lymphocytes		2.41
maturation of lymphocytes		-2.39
chemotaxis of granulocytes	-2.37	
maturation of leukocytes		-2.36
coagulation	-2.36	
Lymphocyte migration		-2.35
necrosis	-1.27	1.07
quantity of helper T lymphocytes		-2.28
infiltration by neutrophils	-2.25	
activation of T lymphocytes		-2.23
size of tumor		-2.22
Fibrosis	-1.51	-0.70
cell death of lymphatic system cells		2.22
immune response of tumor cell lines		-2.20
transformation of fibroblast cell lines		-2.20
morphology of lymph follicle		2.19
cell viability of cancer cells		-2.19
immune response of T lymphocytes		-2.18
morphology of lymphatic system component		2.18
cell death of B lymphocytes		2.18
proliferation of carcinoma cell lines	-2.18	
quantity of natural killer cells		-2.17
phagocytosis of myeloid cells	0.17	-2.00
apoptosis of B-lymphocyte derived cell lines	-1.99	-0.18
recruitment of leukocytes	-0.58	-1.58
chemotaxis of mononuclear leukocytes	-2.16	
cell death of lymphocytes		2.16
apoptosis of B lymphocytes		2.14
binding of granulocytes	2.14	
differentiation of blood cells	2.11	
cell viability of B lymphocytes		-2.11
quantity of IgG3		-2.10
killing of bacteria	-2.08	
quantity of monocytes	2.07	
apoptosis of lymphoid organ	0.55	1.52
organismal death	-2.06	
homing of blood cells	-2.03	
aggregation of cells	-0.58	-1.45
quantity of IgG2b		-2.02

quantity of thymocytes		-2.01
uptake of lipid	2.01	
vascular disease	0.72	-1.28
attachment of cells		-2.00
depletion of cells		2.00
congenital anomaly of digestive system	-1.98	
apoptosis of macrophages	-1.98	
aggregation of blood platelets		-1.98
recruitment of lymphocytes		-1.98
adhesion of lymphocytes		-1.97
T cell migration		-1.97
self-renewal of cells	1.97	
morphology of germinal center		1.95
lack of germinal center		1.95
stimulation of cells	0.19	-1.76
cell movement of monocytes	-1.94	
chemotaxis of monocytes	-1.94	
activation of microglia	-1.94	
quantity of red blood cells	1.94	
quantity of lymphatic system cells	0.89	-1.05
formation of germinal center		-1.93
morbidity or mortality	0.48	1.45
activation of blood platelets	-1.91	
infiltration of leukocytes	-0.94	-0.95
apoptosis of lymphocytes		1.88
migration of mononuclear leukocytes		-1.87
proliferation of blood cells	-0.78	-1.04
quantity of marginal-zone B lymphocytes		-1.81
homing of cells	-0.80	-0.99
cell death of tumor cell lines	-1.05	0.72
quantity of IgA		-1.75
cell viability of lymphocytes		-1.75
infiltration of granulocytes	-1.74	
activation of neutrophils	-1.71	
cleavage of carbohydrate	1.71	
development of tumor		1.71
response of granulocytes	-1.70	
necrosis of epithelial tissue	0.47	1.22
chemotaxis of cells	-1.25	-0.43
proliferation of cells	-0.38	-1.31
cell death of lymphoma cell lines		1.67
hematocrit	1.66	
cell death of macrophages	-1.66	
generation of leukocytes		-1.63
synthesis of reactive oxygen species	-0.21	1.41
degradation of extracellular matrix		1.61
engulfment of cells	1.61	
quantity of IgG2a		-1.60
activation of B lymphocytes		-1.59
binding of blood cells	1.59	
necrosis of tumor	-1.58	
occlusion of artery	0.87	-0.71
mobilization of Ca2+		-1.58
disorder of artery	0.86	-0.71
recruitment of phagocytes		-1.57

cellular homeostasis	1.10	-0.45
homing of lymphocytes		-1.54
apoptosis of tumor cell lines	-1.03	0.51
quantity of steroid	-1.53	
engulfment of antigen presenting cells	1.53	
metabolism of reactive oxygen species	-0.12	1.41
response of mononuclear leukocytes		-1.52
atherosclerosis	0.80	-0.71
cell movement of B lymphocytes		-1.50
proliferation of lung cancer cell lines	-1.49	
mass of fat pad		-1.47
apoptosis of neuroblastoma cell lines	0.73	0.73
accumulation of myeloid cells	-1.46	
vascular lesion		-1.44
quantity of immunoglobulin		-1.43
accumulation of phagocytes	-1.41	
development of blood vessel	-1.11	-0.30
influx of Ca2+		1.41
glomerulonephritis		-1.41
Nephritis		-1.41
engulfment of blood cells		-1.40
proliferation of lymphatic system cells	-1.39	
internalization of cells		-1.39
fatty acid metabolism	1.37	
apoptosis of leukocytes	0.29	1.05
recruitment of granulocytes		-1.34
accumulation of leukocytes	-1.04	0.29
aortic disorder	-0.47	-0.85
chemotaxis of leukocytes		-1.30
generation of lymphocytes		-1.29
formation of lymphatic system component		-1.29
tyrosine phosphorylation of protein		-1.29
quantity of metal	1.17	0.11
binding of cells	1.26	
cell death of immune cells	-0.95	0.30
recruitment of myeloid cells	-0.26	-0.99
differentiation of T lymphocytes		1.25
metastasis	0.60	-0.63
inflammation of body region	-0.39	-0.83
apoptosis of cardiomyocytes	-1.22	
apoptosis of hepatoma cell lines		-1.21
invasion of tumor cells		1.21
binding of tumor cell lines	1.20	
development of cardiovascular system	-1.19	
proteolysis of Gelatin		1.19
phagocytosis by macrophages	1.18	
growth of epithelial tissue	-1.17	
quantity of male germ cells	1.17	
recruitment of eosinophils		-1.17
shape change of leukocytes		-1.17
failure of kidney	-1.15	
quantity of heavy metal	1.15	
apoptosis of lymphoma cell lines		1.15
inflammation of lung	-0.44	0.71
infiltration of cells		-1.14
binding of gonadal cell lines	1.13	

atherosclerotic lesion		-1.13
growth of bacteria	1.13	
concentration of hormone	1.13	
Thrombosis	-1.11	
migration of myeloid cells	1.11	
vasculogenesis	-0.96	-0.14
invasion of tumor		1.10
proliferation of B lymphocytes		-1.10
cleavage of polysaccharide	1.09	
Movement Disorders	-1.09	
accumulation of cells	-1.08	
Lesion Formation	-0.16	0.92
quantity of IgE		-1.08
phagocytosis of blood cells		-1.07
sepsis	1.07	
activation-induced cell death		-1.07
arteriosclerosis	1.07	
cell death of tumor cells		-1.06
apoptosis of cortical neurons		1.06
proliferation of tumor cells	-0.88	0.18
quantity of eosinophils		1.05
survival of organism	-0.01	1.04
differentiation of phagocytes	1.01	
cell death	-0.73	-0.28
activation of regulatory T lymphocytes		-1.00
development of B lymphocytes		-1.00
cytotoxic reaction of cells	-1.00	
accumulation of reactive oxygen species	-1.00	
lymphoproliferative disorder		-1.00
glucose tolerance	-1.00	
growth of lymphatic system component		-1.00
quantity of antigen presenting cells		-1.00
fragmentation of DNA	-0.98	
apoptosis	0.00	0.97
proliferation of T lymphocytes		-0.96
invasion of tissue		0.96
invasion of breast cancer cell lines		0.96
cancer of secretory structure		0.96
cell death of T lymphocytes		0.95
development of lymphocytes		-0.94
differentiation of B lymphocytes		-0.93
quantity of IgM		-0.92
cell movement of endothelial cells		0.90
size of infarct		0.90
quantity of memory T lymphocytes		-0.90
Encephalitis		-0.89
proliferation of fibroblasts	-0.67	0.20
differentiation of cells		-0.87
transmembrane potential of mitochondria		-0.86
proliferation of brain cancer cell lines	-0.84	
cell movement of tumor cell lines		-0.84
glucose metabolism disorder		-0.83
differentiation of hematopoietic progenitor cells		0.82
hyperplasia of spleen	0.00	0.82
inflammation of organ	-0.39	-0.42
inflammation of body cavity	-0.23	0.57

maturation of cells	0.80	
hypersensitive reaction		-0.79
G1 phase	-0.78	
apoptosis of cancer cells		0.78
apoptosis of melanoma cell lines		0.77
differentiation of lymphocytes		0.77
experimental autoimmune encephalomyelitis		-0.76
size of lesion	-0.44	0.31
migration of B lymphocytes		-0.75
cell death of thymocytes	0.74	
proliferation of lymphocytes		-0.73
mass of connective tissue		-0.73
endocytosis	0.72	
neovascularization	-0.71	
proliferation of connective tissue cells		-0.71
cell death of phagocytes	-0.22	-0.49
proliferation of leukemia cell lines	-0.69	
aggregation of blood cells	-0.69	
quantity of plasma cells		0.69
synthesis of nucleotide	0.65	
metabolism of nucleotide	0.65	
synthesis of purine nucleotide	0.65	
quantity of phagocytes		-0.63
apoptosis of T lymphocytes		0.62
apoptosis of epithelial cells	0.13	0.49
cell movement of macrophages	-0.62	
advanced malignant tumor	0.60	
hyperplasia		0.60
apoptosis of hepatocytes		0.60
quantity of cytotoxic T cells		-0.60
growth of tumor	-0.57	-0.02
apoptosis of leukocyte cell lines		-0.59
diabetes mellitus		-0.59
migration of tumor cell lines		-0.58
injury of mice		0.58
quantity of transitional B lymphocytes		0.58
necrosis of liver		0.58
colony formation	-0.56	
mass of heart	-0.55	
extension of plasma membrane projections	0.54	
differentiation of connective tissue cells		0.53
Lymphocyte homeostasis		-0.53
T cell homeostasis		-0.53
T cell development		-0.53
cell death of epithelial cells	0.09	0.43
cell death of myeloid cells	-0.52	
recruitment of neutrophils	-0.51	
quantity of B-1 lymphocytes		-0.50
presence of lymphocytes		-0.49
response of lymphocytes		-0.49
proteolysis	-0.48	
engulfment of myeloid cells	0.48	
quantity of blood platelets	-0.47	
allergy		-0.47
ion homeostasis of cells		-0.46

flux of Ca2+			-0.46
activation of antigen presenting cells			-0.46
blood pressure		0.46	
cell spreading			-0.45
mortality		-0.45	
angiogenesis			0.44
interphase	0.25	0.17	
differentiation of leukocytes			0.43
apoptosis of tumor cells			0.42
apoptosis of stem cells			0.39
hyperplasia of lymphoid organ		0.39	
metabolism of eicosanoid		0.39	
cell viability of connective tissue cells			0.39
quantity of reactive oxygen species		-0.38	
cell movement of vascular smooth muscle cells			-0.37
differentiation of connective tissue			0.36
insulin-dependent diabetes mellitus			-0.36
cell survival		0.34	
development of cardiovascular tissue		-0.34	
damage of lung		-0.34	
activation of tumor cells			-0.33
inflammation of respiratory system component			-0.33
lymphocytic cancer			-0.33
proliferation of immune cells		-0.33	
quantity of granulocytes			0.31
formation of lymphoid organ			0.28
hematologic cancer			0.28
invasion of malignant tumor			-0.28
lymphoid cancer			0.28
quantity of pre-B lymphocytes			0.28
development of lymphatic system component			0.28
proliferation of endothelial cells		-0.28	
migration of fibroblast cell lines		0.27	
cell death of cortical neurons		-0.26	
migration of blood cells		-0.26	
apoptosis of fibroblast cell lines			-0.26
proliferation of tumor cell lines		-0.26	
quantity of connective tissue cells		0.26	
atherogenesis		0.25	
cell death of cardiomyocytes			0.25
inflammation of liver		-0.24	
generation of reactive oxygen species		-0.24	
adhesion of granulocytes		0.22	
clearance of cells		0.22	
stimulation of leukocytes		0.22	
differentiation of mononuclear leukocytes			0.21
damage of liver			-0.20
quantity of neutrophils			-0.18
interphase of tumor cell lines			0.17
phagocytosis of phagocytes		0.17	
apoptosis of leukemia cell lines			0.17
quantity of protein in blood			-0.16
apoptosis of phagocytes		-0.16	
apoptosis of myeloid cells		-0.16	
cell viability		0.15	
cytolysis		-0.15	

response of myeloid cells	-0.15	
migration of tumor cells		-0.15
localization of B lymphocytes		0.15
mean arterial pressure	-0.15	
immediate hypersensitivity		0.15
proliferation of bone marrow cells	-0.15	
degradation of protein	-0.15	
anemia	-0.13	
quantity of Ca2+		-0.13
degradation of DNA		0.12
cell movement of colon cancer cell lines	-0.12	
cell death of heart		-0.11
cell death of connective tissue cells		-0.11
acute coronary syndrome	0.10	
inflammation of respiratory system	-0.10	
cell death of hematopoietic cell lines		-0.10
systemic autoimmune syndrome		0.09
apoptosis of bone cancer cell lines		0.08
sensitivity of cells		0.07
cell death of cerebral cortex cells	-0.05	
endothelial cell development	0.05	
neoplasia of cells		0.05
metabolism of protein	-0.04	
apoptosis of liver cells		0.02
production of reactive oxygen species	0.02	

Supplementary Table S9. IPA pathway analysis of diseases and biological functions in the thyroid

Thyroid			
Irradiated tissues:	Thyroid	Kidneys, liver, lungs, spleen	Kidneys, liver, lungs, spleen, thyroid
**Diseases and Bio Functions**	**Activation z-score**		
size of body	-2.03	1.15	
quantity of carbohydrate		2.21	-0.89
hypertrophy of heart	2.97		
Hypertrophy	2.64		
necrosis of muscle	2.60		
concentration of D-glucose		1.98	-0.60
cell death of muscle cells	2.40		
differentiation of muscle cells	-2.39		
differentiation of muscle	-2.39		
contractility of skeletal muscle	-2.39		
glucose metabolism disorder			-2.39
concentration of lipid		1.84	-0.52
contractility of muscle	-2.35		
differentiation of cells			2.25
cardiomyopathy	2.19		
blood pressure	-2.18		
damage of nervous system			-2.17
insulin resistance			-2.16
respiratory failure	2.00		
hypertrophy of heart chamber	1.98		
hypertrophy of left ventricle	1.98		
differentiation of myoblasts	-1.96		
mass of skeletal muscle	-1.95		
damage of brain			-1.94
inflammation of organ			-1.93
mass of adipose tissue			1.88
binding of DNA			1.75
perinatal death	1.64		
contractility of cardiac muscle	-1.40		
ingestion by mice			-1.39
contractility of ventricular myocardium	-1.39		
thermoregulation			1.38
neonatal death	1.37		
development of cardiovascular system	-1.32		
myopathy	1.19		
cellular homeostasis			1.18
metabolism of triacylglycerol			1.18
synthesis of triacylglycerol			1.18
accumulation of lipid			1.13
obesity			1.11
Edema		1.10	
respiratory quotient			1.07
body temperature			-1.07
consumption of oxygen			-1.06

differentiation of adipocytes			1.05
synthesis of reactive oxygen species			-1.04
muscle contraction	1.03		
quantity of insulin in blood			-1.00
contraction of heart	0.91		
quantity of adipose tissue			0.89
cyanosis	0.85		
cell death of epithelial cells			0.83
Fibrosis	0.81		
quantity of leptin in blood			0.81
production of reactive oxygen species			-0.80
synthesis of lipid			0.80
behavior			-0.80
contraction of striated muscle	0.76		
insulin sensitivity			-0.76
quantity of protein in blood			0.54
hepatic steatosis			-0.52
feeding			-0.49
concentration of hormone			0.45
quantity of white adipose tissue			0.45
mass of heart	-0.45		
concentration of triacylglycerol			-0.38
size of cells	-0.34		
morphology of cells	-0.34		
mass of organism			0.25
apoptosis of connective tissue cells			0.22
glucose tolerance			0.17
synthesis of fatty acid			0.11
concentration of fatty acid			0.04
microtubule dynamics		0.03	
concentration of cholesterol			0.03
heart rate	0.01		

SUPPLEMENTARY TABLE S10. List of ionizing radiation-associated genes

Gene symbol (*Mus musculus*)	Synonym(s)	Known RNA expression of human homolog*				
		Kidney	Liver	Lung	Spleen	Thyroid
		Expression (FPKM)				
Amy2a5	*Amy2, Amy-2*	15	9	18	19	17
Apaf1	*Apaf1l*	5	4	7	10	5
Bag1	*Rap46*	84	31	52	38	54
Batf3		1	1	4	10	1
Bax		39	27	51	77	26
Birc2	*Api1, cIAP1, HIAP1, IAP1, MIAP1, MIHB*	24	27	23	25	23
Birc3	*Api2, cIAP2, HIAP2, IAP2, MIAP2, MIHC, RNF49*	6	19	16	58	3
Ccnb1	*Ccnb1-rs13, Cycb-4*	4	3	5	8	10
Ccnd1	*bcl-1, cD1, CycD1*	36	91	72	37	51
Ccne1	*CycE1*	1	1	1	2	2
Ccng1		101	73	37	36	74
Cdc25c	*Cdc25*	0	1	0	0	0
Cdkn1a	*CAP20, CDKI, P21*	18	58	36	11	44
Akr1c21		26	116	13	1	4
Ddb2	*p48*	5	16	14	13	6
Ephx2	*Eph2, sEH, sEP*	139	132	14	14	25
Fen1		5	6	5	8	5
Fhl2	*SLIM3*	18	15	8	13	21
Flt3	*CD135, Flk2, wmfl*	2	0	1	8	0
Fos	*cFos, D12Rfj1*	288	223	444	153	676
Gadd45a	*Ddit1*	68	116	14	10	47
Gadd45g	*CR6, OIG37*	8	36	5	1	3
Gja1	*Cx43*	14	8	63	3	144
Gjb2	*Cx26*	8	28	1	0	2
Irf8	*ICSBP, Icsbp1, IRF-8, Myls*	5	14	21	113	3
Jun	*c-jun, Junc*	80	127	72	78	132
Mapk8ip1	*IB1, Jip1, MAPK8IP1, mjip-2a, Prkm8ip, Skip*	6	2	2	1	3
Mapk1	*Erk2, MAPK2, p42mapk, Prkm1*	40	23	38	40	42
Mapk3	*Erk1, Esrk1, Mtap2k, p44erk1, p44mapk, Prkm3*	47	12	81	52	70
Mdm2		12	30	18	18	21
Mycbp	*Amy-1*	19	8	15	12	18
Naa35	*Mak10*	11	5	8	7	16
Pmaip1	*Noxa*	1	1	2	5	3
Ogg1	*Mmh*	32	7	11	13	11
Pcna		40	31	35	40	40
Plcg2		25	13	12	47	10
Rad23b	*mHR23B*	54	120	50	43	66
Rad51b	*mREC2, R51H2, Rad51l1*	6	2	5	4	12
Rhoa	*Arha, Arha1, Arha2, RhoA*	204	128	346	257	280
Rhoh	*Arhh*	1	2	4	36	1
Tgfb3		4	7	13	5	7
Tgfbr2	*TbetaRII, TBR-II*	77	33	152	104	62
Tnfaip8	*Gg2-1, Gm10539, Nded, Ssc-2*	11	6	16	57	23
Tnfrsf10b	*DR5, KILLER, TRAILR2, TRICK2A, TRICK2B, TRICKB*	15	28	27	25	25
Tnfrsf19	*TAJ, TAJ-ALPHA, TRADE, Troy*	15	0	19	5	3
Tnfrsf21	*DR6, TR7*	39	6	13	15	5
Trp53	*p53*	12	9	15	28	17
Trp53inp1	*SIP, Teap*	10	43	21	29	8
Trp53inp2	*Tp53inp2*	22	27	13	5	18
Vegfb	*Vrf*	62	20	54	44	50
Wee1		12	33	13	9	34
Xab2		18	7	17	26	18
Xiap	*Aipa, Api3, Birc4, IAP3, ILP-1*	15	9	11	11	18
Xpc		27	15	22	23	28

Xrcc1		16	7	19	19	25
Xrcc6	*Ku70, G22p1*	85	63	72	71	83

*according to Human Protein Atlas (http://www.proteinatlas.org; retrieved 2014-08-14)
FPKM, fragments per kilobase of exon per million fragments mapped

The table has been adapted from:

Langen B. (2015). Systemic effects after ionizing radiation exposure: Genome-wide transcriptional analysis of mouse normal tissues exposed to ^{211}At, ^{131}I, or 4 MV photon beam (Doctoral dissertation). Chalmers University of Technology, Gothenburg, Sweden (ISBN 978-91-7597-194-0).

The list is composed of data presented in:

Snyder AR, Morgan WF. Gene expression profiling after irradiation: clues to understanding acute and persistent responses? *Cancer Metastasis Rev.* 2004;23:259–268.
Chaudhry MA. Biomarkers for human radiation exposure. *J Biomed Sci*. 2008;15:557–563.

SUPPLEMENTARY TABLE S11. List of thyroid hormone-responding genes

Gene symbol (*Mus musculus*)	Synonym(s), comment	Reference	Known RNA expression of human homolog* Kidney	Liver	Lung	Spleen	Thyroid
			Expression (FPKM)				
Alpl	*Akp2, TNAP*	Moeller *et al.* (2011)	55	29	51	12	3
Akt1†	*Akt, PKB*	Cordeiro *et al.* (2013)	41	39	77	70	55
Angptl3	*hypl*	Moeller *et al.* (2011)	45	313	0	0	0
Atp2a1†	*SERCA1*	Kim (2008)	1	0	1	1	0
Atp5c1		Pihlajamäki *et al.* (2009)	250	146	89	92	157
Camkk1†		Cordeiro *et al.* (2013)	2	1	4	5	7
Camkk2†		Cordeiro *et al.* (2013)	15	8	20	28	13
Ccnd1	*bcl-1, cD1, CycD1*	Puzianowska-Kuznicka *et al.* (2006)	36	91	72	37	51
Cd44	*Ly-24, HERMES, Pgp-1*	Dong *et al.* (2009)	17	21	184	72	84
Cdkn1a	*CAP20, CDKI, P21*	Puzianowska-Kuznicka *et al.* (2006)	18	58	36	11	44
Cox7c	*Cox7c1, COXVIIc*	Pihlajamäki *et al.* (2009)	389	235	173	187	279
Cyp7a1†		Cordeiro *et al.* (2013)	0	17	0	0	0
E2f1		Puzianowska-Kuznicka *et al.* (2006)	0	1	3	2	1
Egf		Puzianowska-Kuznicka *et al.* (2006)	95	0	1	0	4
Egfr		Puzianowska-Kuznicka *et al.* (2006)	11	22	16	5	23
Epas1	*bHLHe73, HIF2A, HLF, HRF*	Moeller *et al.* (2011)	97	82	432	82	146
Fas†	*APO-1, CD95, TNFR6, Tnfrsf6*	Cordeiro *et al.* (2013)	9	24	24	16	10
Fgf2	*bFGF, Fgf-2, Fgfb*	Moeller *et al.* (2011)	4	1	5	1	2
Fos	*cFos, D12Rfj1*	Puzianowska-Kuznicka *et al.* (2006)	288	223	444	153	676
Fosb		Puzianowska-Kuznicka *et al.* (2006)	30	93	44	19	179
Furin	*Fur, PACE, Pcsk3, SPC1*	Moeller *et al.* (2011)	25	66	32	26	40
G6pc	*G6Pase, G6pt*	Lin *et al.* (2013)	54	136	0	0	0
Gsta1	(human homolog *GSTA1* as representative of RNA expression in tissue	Lin *et al.* (2013)	739	1020	14	0	0
Gtf3c1		Dong *et al.* (2009)	18	9	17	16	33
Hadha†	*Mtpa*	Chocron *et al.* (2012)	97	97	51	52	66
Hif1a	*bHLHe78, MOP1*	Lin *et al.* (2013); Moeller *et al.* (2011)	70	35	40	65	55
Hnrnph3	*hnRNP, 2H9, Hnrph3*	Pihlajamäki *et al.* (2009)	94	56	98	112	123
Ifi27	*Ifi27l1*	Puzianowska-Kuznicka *et al.* (2006)	73	39	388	480	52
Ldlr†		Cordeiro *et al.* (2013)	6	62	52	11	3
Lmo2	*Rbtn2, Rhom2*	Dong *et al.* (2009)	31	7	35	35	12
Mag	*Gma, siglec-4a*	Dong *et al.* (2009)	1	1	1	0	1
Mapk1†	*Erk2, MAPK2, Prkm1*	Cordeiro *et al.* (2013)	40	23	38	40	42
Mbp	*golli-mbp, Hmbpr*	Dong *et al.* (2009)	21	12	22	26	26
Mcl1		Moeller *et al.* (2011)	95	262	162	174	168
Mdm2		Puzianowska-Kuznicka *et al.* (2006)	12	30	18	18	21
Myh6	*alpha-MHC, alpha myosin, Myhca*	Moeller *et al.* (2011)	0	0	0	0	0
Ncor1†	*N-CoR, mKIAA1047, Rxrip13*	Söderström *et al.* (1997); Cheng *et al.* (2010)	34	57	28	34	40
Ncor2†	*SMRT*	Söderström *et al.* (1997); Cheng *et al.* (2010)	22	9	40	34	18
Nos1†	*bNOS, nNOS, NO, Nos-1*	Carreras *et al.* (2001)	2	0	1	0	0
Pck1	*PEPCK*	Lin *et al.* (2013)	490	687	0	0	0

Pfkp	*PFK-C*	Moeller *et al.* (2011)	33	3	13	21	33
Pik3ca†	*caPI3K, p110alpha*	Cordeiro *et al.* (2013)	11	7	12	13	11
Prka† *family*	human homolog *PRKAA1* as representative of RNA expression in tissue	Cordeiro *et al.* (2013)	35	34	36	35	43
Prkc† *family*	human homolog *PRKCA* as representative of RNA expression in tissue	Cordeiro *et al.* (2013)	14	3	6	7	4
Psma1	*C2, HC2, Pros-30*	Pihlajamäki *et al.* (2009)	79	66	85	83	98
Psmd12	*P55*	Pihlajamäki *et al.* (2009)	21	25	15	17	21
Rcan2	*Csp2, Dscr1l1, MCIP2, ZAKI-4*	Moeller *et al.* (2011)	32	4	16	8	32
Rxr† *family*	human homolog *RXRA* as representative of RNA expression in tissue	Cordeiro *et al.* (2013)	27	28	28	32	27
Slc16a4		Moeller *et al.* (2011)	113	12	11	3	12
Slc16a6		Lin *et al.* (2013)	1	1	2	5	2
Slc2a1	*Glut1*	Moeller *et al.* (2011)	13	2	4	11	9
Slc2a4	*Glut4*	Brunetto *et al.* (2012)	2	1	1	0	2
Sms	*SpmST*	Dong *et al.* (2009)	27	8	7	10	9
Snrpe		Pihlajamäki *et al.* (2009)	11	8	7	7	6
Srebf1†	*ADD-1, bHLHd1, SREBP1, SREBP-1a, SREBP1c*	Cordeiro *et al.* (2013)	27	63	51	26	31
Stc1		Moeller *et al.* (2011)	12	4	5	0	28
Tab1	*Map3k7ip1*	Puzianowska-Kuznicka *et al.* (2006)	9	6	10	13	8
Thra†	*Erba, Nr1a1, Rvr, Thra1, Thra2*	Ribeiro (2008)	27	3	27	13	25
Thrb†	*c-erbAbeta, Nr1a2, T3Rbeta, Thrb1, Thrb2*	Ribeiro (2008)	13	14	7	6	6
Thrsp	*S14, Spot 14*	Moeller *et al.* (2011)	2	143	0	0	5
Vldlr		Dong *et al.* (2009)	13	1	13	8	15

*according to Human Protein Atlas (http://www.proteinatlas.org; retrieved 2014-08-27)

†literature reference (analysis) on protein

FPKM, fragments per kilobase of exon per million fragments mapped

The table has been adapted from:

Langen B. (2015). Systemic effects after ionizing radiation exposure: Genome-wide transcriptional analysis of mouse normal tissues exposed to ^{211}At, ^{131}I, or 4 MV photon beam (Doctoral dissertation). Chalmers University of Technology, Gothenburg, Sweden (ISBN 978-91-7597-194-0).

The list is composed of data presented in:

Carreras MC, Peralta JG, Converso DP, Finocchietto PV, Rebagliati I, Zaninovich AA, Poderoso JJ. Modulation of liver mitochondrial NOS is implicated in thyroid-dependent regulation of O(2) uptake. *Am J Physiol Heart Circ Physiol*. 2001;281(6):H2282–8.

Chocron ES, Sayre NL, Holstein D, Saelim N, Ibdah JA, Dong LQ, Zhu X, Cheng SY, Lechleiter JD. The trifunctional protein mediates thyroid hormone receptor-dependent stimulation of mitochondria metabolism. *Mol Endocrinol*. 2012;26(7):1117–28.

Ribeiro MO. Effects of thyroid hormone analogs on lipid metabolism and thermogenesis. *Thyroid*. 2008;18(2):197–203.

Kim B. Thyroid hormone as a determinant of energy expenditure and the basal metabolic rate. *Thyroid*. 2008;18(2):141–4.

Cordeiro A, Souza LL, Einicker-Lamas M, Pazos-Moura CC. Non-classic thyroid hormone signalling involved in hepatic lipid metabolism. *J Endocrinol*. 2013 Jan 7. [Epub ahead of print]

Söderström M, Vo A, Heinzel T, Lavinsky RM, Yang WM, Seto E, Peterson DA, Rosenfeld MG, Glass CK. Differential effects of nuclear receptor corepressor (N-CoR) expression levels on retinoic acid receptor-mediated repression support the existence of dynamically regulated corepressor complexes. *Mol Endocrinol*. 1997;11(6):682–92.

Cheng SY, Leonard JL, Davis PJ. Molecular aspects of thyroid hormone actions. *Endocr Rev*. 2010;31(2):139–70.

Lin JZ, Sieglaff DH, Yuan C, Su J, Arumanayagam AS, Firouzbakht S, Cantu Pompa JJ, Reynolds FD, Zhou X, Cvoro A, Webb P. Gene specific actions of thyroid hormone receptor subtypes. *PLoS One*. 2013;8(1):e52407.

Moeller LC, Broecker-Preuss M. Transcriptional regulation by nonclassical action of thyroid hormone. *Thyroid Res*. 2011;4 Suppl 1:S6.

Brunetto EL, Teixeira Sda S, Giannocco G, Machado UF, Nunes MT. T3 rapidly increases SLC2A4 gene expression and GLUT4 trafficking to the plasma membrane in skeletal muscle of rat and improves glucose homeostasis. *Thyroid*. 2012;22(1):70–9.

Puzianowska-Kuznicka M, Pietrzak M, Turowska O, Nauman A. Thyroid hormones and their receptors in the regulation of cell proliferation. *Acta Biochim Pol*. 2006;53(4):641–50.

Pihlajamäki J, Boes T, Kim EY, Dearie F, Kim BW, Schroeder J, Mun E, Nasser I, Park PJ, Bianco AC, Goldfine AB, Patti ME. Thyroid hormone-related regulation of gene expression in human fatty liver. *J Clin Endocrinol Metab*. 2009;94(9):3521–9.

Dong H, Yauk CL, Rowan-Carroll A, You SH, Zoeller RT, Lambert I, Wade MG. Identification of thyroid hormone receptor binding sites and target genes using ChIP-on-chip in developing mouse cerebellum. *PLoS One*. 2009;4(2):e4610.

				Irradiated tissue		
				Thyroid (group A)	Kidneys, liver, lungs, spleen (group B)	Kidneys, liver, lungs, spleen, thyroid (group C)
Tissue	Gene symbol	Probe ID	Transcript ID	Differential regulation (log_2 ratio)		
Liver	*Alb*	ILMN_2651160	ILMN_214721	-1.39	-1.14	-0.73
	Fgf21	ILMN_2710698	ILMN_219576	0.59	0.86	1.00
	Mup4	ILMN_2592166	ILMN_209061	-0.83	-0.64	-0.81
	OTTMUSG00000007485	ILMN_2881950	ILMN_241785	-1.24	-0.99	-0.89
	Rgn	ILMN_2700885	ILMN_218841	-0.74	-0.70	-0.84
	Serpina4-ps1	ILMN_2815450	ILMN_245485	-1.86	-0.74	-1.53
Kidney medulla	*1600029D21Rik*	ILMN_1259777	ILMN_222410	1.03	1.06	1.09
	Aadat	ILMN_2594302	ILMN_209280	0.63	1.00	0.67
	Acin1	ILMN_2755195	ILMN_222814	1.95	1.93	1.29
	Afmid	ILMN_2827607	ILMN_209564	-0.65	-0.67	-0.83
	Aifm1	ILMN_2881272	ILMN_218675	0.99	1.02	0.73
	Akp2	ILMN_2661287	ILMN_215583	-0.60	-0.68	-0.80
	Akr1b3	ILMN_2591917	ILMN_240453	1.26	1.18	1.05
	Arg2	ILMN_2669164	ILMN_216267	0.73	0.71	1.18
	Asl	ILMN_2670542	ILMN_215677	-0.59	-0.59	-0.93
	Atp5a1	ILMN_2633229	ILMN_213071	1.08	0.99	0.86
	Bst1	ILMN_2648346	ILMN_214474	0.65	0.73	0.68
	Btg1	ILMN_2866189	ILMN_212740	0.75	0.65	0.86
	C1qtnf3	ILMN_2863849	ILMN_209151	-0.59	-0.58	-0.89
	Cox7b	ILMN_2671689	ILMN_242680	1.30	1.27	0.92
	Ctnnb1	ILMN_2994460	ILMN_218498	0.99	0.64	0.79
	Cyfip2	ILMN_2670713	ILMN_214228	1.16	1.11	0.86
	Cyp1b1	ILMN_1232716	ILMN_252141	0.81	0.84	1.01
	Cyp2d26	ILMN_1227936	ILMN_213983	-0.68	-0.81	-0.94
	Cyp2d26	ILMN_2642975	ILMN_213983	-0.59	-0.70	-0.75
	Cyp4b1	ILMN_2790496	ILMN_218342	-0.68	-0.91	-0.71
	Ddx3y	ILMN_2899599	ILMN_214603	0.77	0.81	0.67
	Dhx15	ILMN_1218571	ILMN_219910	0.73	0.73	0.72
	Dld	ILMN_2703657	ILMN_219055	0.99	0.84	0.79
	Ednrb	ILMN_2917471	ILMN_208799	1.01	0.70	0.96
	Eed	ILMN_2765015	ILMN_223515	0.73	0.65	0.66
	Eef1a1	ILMN_1244894	ILMN_220931	1.38	1.40	0.99
	Eef1b2	ILMN_2874853	ILMN_212511	0.97	0.85	0.91
	G6pc	ILMN_1245103	ILMN_219085	-0.68	-0.84	-1.08
	Gabrb3	ILMN_3120335	ILMN_240761	-0.67	-0.60	-1.06
	Gamt	ILMN_2815506	ILMN_222965	-0.65	-0.58	-0.84
	Gjb2	ILMN_1227148	ILMN_210347	0.75	0.70	0.64
	Gpm6a	ILMN_2909336	ILMN_217544	1.03	1.01	0.95
	Hbb-b1	ILMN_1235372	ILMN_212995	0.93	0.80	0.84
	Hnrpdl	ILMN_2903440	ILMN_217979	1.04	0.93	1.01
	Hpd	ILMN_2731444	ILMN_221134	-1.30	-1.11	-1.33

Hrsp12	ILMN_1251300	ILMN_210108	0.87	0.91	0.67
Hsp90aa1	ILMN_2752883	ILMN_215698	0.96	0.70	0.76
Ifit2	ILMN_2981169	ILMN_216122	0.76	0.66	0.92
Itm2b	ILMN_2782082	ILMN_216376	0.68	0.60	0.77
Kcnj1	ILMN_2924677	ILMN_209494	0.86	0.77	0.66
Klk1b27	ILMN_1252131	ILMN_196774	-0.80	-0.78	-0.83
Kpnb1	ILMN_1230411	ILMN_219048	0.79	0.80	0.92
Lhfp	ILMN_2879588	ILMN_212576	0.75	0.62	0.67
LOC100041703	ILMN_3163159	ILMN_312763	1.29	1.24	1.00
LOC100044087	ILMN_1237846	ILMN_318899	0.98	1.07	0.79
LOC100044779	ILMN_2755660	ILMN_312441	1.29	1.43	1.26
LOC100047634	ILMN_1215644	ILMN_309955	0.67	0.67	0.68
LOC100048480	ILMN_2497616	ILMN_310214	1.08	0.76	0.83
LOC546015	ILMN_1214918	ILMN_310507	0.98	1.15	1.01
LOC667250	ILMN_3123441	ILMN_244603	1.04	1.24	0.97
LOC668837	ILMN_2587859	ILMN_312076	1.46	1.53	1.06
Lypla1	ILMN_2873319	ILMN_217050	0.94	1.16	0.83
Mal	ILMN_1248947	ILMN_211935	0.98	0.77	0.93
Mat2a	ILMN_1258415	ILMN_261868	0.72	0.99	0.73
Mbnl2	ILMN_1254987	ILMN_237158	0.87	0.71	0.93
Mdfic	ILMN_2704637	ILMN_219128	0.84	0.75	0.78
Mdh1	ILMN_2631021	ILMN_212852	1.32	1.34	0.81
Mep1a	ILMN_2616479	ILMN_211502	0.78	1.01	0.81
Myo5b	ILMN_2610576	ILMN_210919	0.60	0.69	0.60
Nab1	ILMN_2721761	ILMN_210731	0.84	0.62	0.81
Nampt	ILMN_2821850	ILMN_212748	0.68	0.73	0.95
Ndufb9	ILMN_2605004	ILMN_210376	1.06	1.16	0.83
Oxr1	ILMN_2697390	ILMN_213669	0.79	0.61	0.79
Pabpc1	ILMN_1259482	ILMN_201529	0.72	0.74	0.76
Pax8	ILMN_1256173	ILMN_211489	0.77	0.66	0.76
Pgam2	ILMN_2588815	ILMN_208713	-0.63	-0.63	-1.04
Pgrmc1	ILMN_2623216	ILMN_212144	0.76	0.72	0.77
Pls3	ILMN_2764781	ILMN_223492	0.96	0.80	0.83
Pmpcb	ILMN_2640122	ILMN_258836	0.97	0.95	0.76
Ppap2a	ILMN_1222991	ILMN_209701	0.64	0.59	0.72
Prodh2	ILMN_1252471	ILMN_209754	-0.63	-0.84	-0.83
Prodh2	ILMN_2598892	ILMN_209754	-0.60	-0.73	-0.95
Psca	ILMN_2713055	ILMN_219740	0.62	1.16	0.65
Ptger3	ILMN_2770866	ILMN_219812	1.00	0.76	0.83
Ptgs1	ILMN_1224866	ILMN_213920	0.75	0.61	0.63
Pthr1	ILMN_2850309	ILMN_213298	-0.72	-0.71	-0.82
Pthr1	ILMN_2650356	ILMN_314683	-0.68	-0.66	-0.85
Ptp4a2	ILMN_2725835	ILMN_210989	0.83	0.75	0.84
Pvalb	ILMN_1218223	ILMN_213948	-0.67	-0.69	-1.41
Rab9	ILMN_2783918	ILMN_213409	0.98	1.01	0.91
Rps3a	ILMN_2733330	ILMN_221275	0.90	1.03	0.85
S100a10	ILMN_1256702	ILMN_211394	0.72	0.72	0.79
Sdhd	ILMN_1239143	ILMN_209676	1.11	1.24	0.86
Sepp1	ILMN_1247553	ILMN_257911	1.05	1.25	0.98
Sh3bgrl	ILMN_2636212	ILMN_213353	0.82	0.87	0.75

Slc11a1	ILMN_2675811	ILMN_254692	1.17	1.14	0.97
Slc13a3	ILMN_2918312	ILMN_220477	-0.61	-0.82	-1.08
Slc14a2	ILMN_3051931	ILMN_245991	1.04	0.76	0.85
Slc26a4	ILMN_1220193	ILMN_209376	-0.61	-0.88	-0.91
Slc38a2	ILMN_2955671	ILMN_217242	1.27	0.90	1.33
Slc39a5	ILMN_3150519	ILMN_261006	-0.65	-0.75	-0.65
Snx1	ILMN_2605465	ILMN_210423	0.96	1.06	0.82
Sord	ILMN_2758198	ILMN_223021	-0.64	-0.76	-0.95
Spp1	ILMN_2690603	ILMN_218026	0.70	0.74	0.70
Ss18	ILMN_1233813	ILMN_242116	0.87	0.76	0.71
Thnsl2	ILMN_3147331	ILMN_246727	-0.70	-0.66	-0.98
Trp53inp1	ILMN_2971479	ILMN_194391	0.75	0.89	0.90
Txn1	ILMN_3009572	ILMN_192535	0.70	0.96	0.82
Ucp1	ILMN_2994373	ILMN_187828	-0.73	-0.80	-0.60
Usmg5	ILMN_1219002	ILMN_194875	1.14	1.15	0.86
Vbp1	ILMN_1243095	ILMN_185209	1.17	0.85	0.95

Supplementary Figure S1. Transcripts regulated at all irradiation setups. Positive numbers indicate upregulation (green), negative numbers indicate downregulation (red).

			Irradiated tissues:	Thyroid (group A)	Kidneys, liver, lungs, spleen (group B)	Kidneys, liver, lungs, spleen, thyroid (group C)	Thyroid (group A)
				Differential regulation (log_2 ratio)			
Tissue	Gene symbol	Probe ID	Transcript ID	in each non-thyroid tissue			in thyroid
Kidney cortex	*S3-12*	ILMN_2588249	ILMN_208656	*ND*	0.75	1.06	-1.97
	Car3	ILMN_2630047	ILMN_212767	*ND*	1.32	1.32	-1.98
	Asns	ILMN_2643513	ILMN_213406	*ND*		0.64	1.93
Kidney medulla	*Pvalb*	ILMN_1218223	ILMN_213948	-0.67	-0.69	-1.41	-2.42
	Pgam2	ILMN_2588815	ILMN_208713	-0.63	-0.63	-1.04	-2.19
	Fkbp11	ILMN_1224635	ILMN_210599	*ND*	*ND*	-0.68	1.95
	Klk1b27	ILMN_1252131	ILMN_196774	-0.80	-0.78	-0.83	2.43
	S3-12	ILMN_2588249	ILMN_208656	*ND*	*ND*	0.70	-1.97
	Schip1	ILMN_2937320	ILMN_216237	*ND*	*ND*	0.74	-1.72
	Gjb2	ILMN_1227148	ILMN_210347	0.75	0.70	0.64	1.64
	Pof1b	ILMN_1242912	ILMN_221065	0.60	*ND*	*ND*	1.20
Liver	*Car3*	ILMN_2630047	ILMN_212767	*ND*	*ND*	-0.88	-1.98
	S3-12	ILMN_2588249	ILMN_208656	*ND*	*ND*	1.84	-1.97
	Mup5	ILMN_3158509	ILMN_214274	-1.04	*ND*	*ND*	2.38
	Asns	ILMN_2643513	ILMN_213406	0.65	*ND*	*ND*	1.93
	Krt23	ILMN_2671165	ILMN_216445	0.58	*ND*	*ND*	2.24
Lungs	*S3-12*	ILMN_2588249	ILMN_208656	*ND*	*ND*	1.78	-1.97
	Car3	ILMN_2630047	ILMN_212767	*ND*	*ND*	1.79	-1.98
	Calca	ILMN_2744927	ILMN_192774	*ND*	*ND*	0.90	2.77
	Myl1	ILMN_2878542	ILMN_218373	*ND*	-1.13	*ND*	-2.41
	Mb	ILMN_2954987	ILMN_210416	*ND*	-0.99	*ND*	-2.26
Spleen	*Aqp1*	ILMN_2980661	ILMN_216314	*ND*	-0.60	*ND*	-1.83
	Csprs	ILMN_2661289	ILMN_215584	*ND*	*ND*	-0.64	1.53
	Kcnn4	ILMN_2765032	ILMN_223516	*ND*	-0.64	*ND*	1.51

Supplementary Figure S2. Shared transcript regulation among thyroid and other tissues at differential exposures. Positive numbers indicate upregulation (green), negative numbers indicate downregulation (red); ND, none detected
